# Multi-Spectroscopic and Molecular Modeling Studies of Interactions Between Anionic Porphyrin and Human Serum Albumin

**DOI:** 10.3390/ijms252212473

**Published:** 2024-11-20

**Authors:** Tadeusz Strózik, Marian Wolszczak, Maria Hilczer, Magdalena Pawlak, Tomasz Wasiak, Piotr Wardęga, Maksim Ionov, Maria Bryszewska

**Affiliations:** 1 Department of Molecular Biology, Medical University of Lodz, Zeligowskiego 7/9 St., 90-752 Lodz, Poland; tomasz.wasiak@umed.lodz.pl; 2 Institute of Applied Radiation Chemistry, Faculty of Chemistry, Lodz University of Technology, 93-590 Lodz, Poland; marian.wolszczak@p.lodz.pl (M.W.); maria.hilczer@p.lodz.pl (M.H.); magdalena.pawlak.2017@gmail.com (M.P.); 3 NanoTemper Technologies sp. z.o.o., Bobrzynskiego 14, 30-348 Krakow, Poland; piotr.wardega@nanotempertech.com; 4 Department of General Biophysics, Faculty of Biology and Environmental Protection, University of Lodz, 141/143 Pomorska St. Building D, 90-236 Lodz, Poland; maksim.ionov@biol.uni.lodz.pl (M.I.); maria.bryszewska@biol.uni.lodz.pl (M.B.); 5 Faculty of Medicine, Collegium Medicum, Mazovian Academy in Plock, Pl. Dabrowskiego 2, 09-402 Plock, Poland

**Keywords:** HSA, TSPP, photosensitizer, Scatchard formalism, binding sites, binding affinity, circular dichroism, T-T absorption, thermal unfolding, molecular docking

## Abstract

The subject of this study is the interaction between 5,10,15,20-tetrakis (4-sulfonatophenyl)–porphyrin (TSPP), a potential photosensitizer for photodynamic therapy (PDT) and radiotherapy, and human serum albumin (HSA), a crucial protein in the body. The main objective was to investigate the binding mechanisms, structural changes, and potential implications of these interactions for drug delivery and therapeutic applications. Spectroscopic techniques and computational methods were employed to investigate the mechanism and effects of TSPP binding by HSA. The results suggest the possibility of simultaneous binding of three TSPP ions at binding sites of different affinity within albumin. The estimated values of the binding constant *K_b_* for these sites were in the range of 0.6 to 6.6 μM^−1^. Laser flash photolysis indicated the stabilization of TSPP in the HSA structure, which resulted in prolonged lifetimes of the excited states (singlet and triplet) of porphyrin. Circular dichroism analysis was used to assess the changes in the secondary and tertiary structures of HSA upon TSPP binding. An analysis of the molecular docking results allowed us to identify the preferred TSPP binding sites within HSA and provided information on the specific interactions of amino acids involved in the stabilization of TSPP–HSA complexes. The estimated free energy of the binding of porphyrin at the three most favorable docking sites found in the HSA structure that was considered native were in the range of −80 to −41 kcal/mol. Finally, thermal unfolding studies showed that TSPP increased the stability of the secondary structure of albumin. All these findings contribute to the understanding of the interactions between TSPP and HSA, offering valuable insights for the development of novel cancer therapy approaches.

## 1. Introduction

Porphyrins belong to the class of cyclic tetrapyrroles that forms a highly conjugated macrocycle. The presence of 18 π-electrons delocalized over the macrocycle gives rise to their long wavelength absorption [1], high molar absorption coefficients, and numerous potentially useful photochemical and photophysical properties [2]. As a result, due to their application as photosensitizing agents (PSs) in medicine [3], especially in photodynamic therapy (PDT) [4,5] as well as in radiotherapy [6], porphyrins have attracted the attention of researchers. As one of the second-generation photosensitizers, 5,10,15,20-tetrakis(4-sulfonatophenyl)porphyrin (TSPP) may be used in PDT [7]. This compound has already been tested for the treatment of cancer [8,9,10].

Porphyrins taken up by the blood circulation system are transported to target tissues by human serum albumin (HSA) and other plasma proteins, such as low- and high-density lipoproteins [11]. HSA is the most abundant plasma protein and plays a crucial role in the transport, distribution, and metabolism of many endogenous and exogenous ligands, such as fatty acids, metabolites, and drugs [12]. In certain cases, the interaction between a ligand and HSA may be highly specific, making it possible to identify the ligand binding sites, although binding may induce some conformational changes in the protein structure [13,14]. The main regions of ligand binding to HSA are located in the hydrophobic cavities of subdomains IIA (binding site I) and IIIA (binding site II). These binding sites are known as the Sudlow I and Sudlow II sites [15]. Other albumin subdomains can also take part in the binding and transport of ligands. One such example is heme, a porphyrin which interacts with subdomain IB [16]. Human serum albumin has a single tryptophan residue buried in the crevice of domain IIA, and the sensitivity of this residue to local changes in the polarity of the medium makes it a useful fluorescent probe.

The binding of porphyrin to HSA has a pronounced effect on its distribution, free concentration, and metabolism [17]. In some cases, this may even significantly affect the photodynamic activity of porphyrin [17,18]. It has been shown that increasing the concentration of TSPP in in vivo studies enhanced its toxic effect [19]. Therefore, understanding the interaction between porphyrin and HSA is of the utmost importance in formulating safe drugs and effective dosages [20,21].

To date, the interaction of porphyrins with serum albumins has been the subject of extensive studies [22,23]. The literature has shown that binding to HSA changes porphyrins’ photophysical and photochemical properties due to their interactions with the specific amino acids near the binding site [20]. It has also been shown that negatively charged porphyrins bind strongly to serum albumins. Investigators have used a wide variety of techniques to study the binding of water-soluble porphyrins to protein. They have applied UV–VIS, fluorescence, circular dichroism (CD) spectroscopies [24], picosecond emission anisotropy, femtosecond emission, and flash photolysis [25]. We have shown how the interaction with HSA affects the absorbance and fluorescence spectra of TSPP [26]. The Soret band of the absorption spectrum of TSPP in buffer solution at pH 7 undergoes a 7 nm redshift upon the addition of HSA. This band corresponds to a strong electronic transition from S_0_ to the second excited state, S_2_. The presence of protein results in a significant rise in porphyrin’s fluorescence intensity; the first and the second maximum of the fluorescence band, recorded at an excitation wavelength of 533 nm, is 2.6 and 3.4 times larger, respectively, for the TSPP + HSA system than for the free TSPP. Changes observed in both the absorption and fluorescence spectra provide evidence of the penetration of anionic porphyrin into the protein structure. However, an issue that is important but has not been fully elucidated so far is the number and structure of the anionic porphyrin binding sites in albumin. Information about these specific sites, as well as the nature of the interactions that bind porphyrin to albumin, may be valuable for understanding the distribution mechanism of porphyrins (drugs) in the human body.

In this paper, we present the results of our study on the binding process of a water-soluble porphyrin, TSPP, to albumin. The study was conducted using various spectroscopic measurement techniques (UV−VIS absorption, laser flash photolysis, circular dichroism, differential scanning fluorimetry) and computer simulation techniques (ab initio calculations, molecular docking, molecular dynamics). We found that the binding of TSPP to HSA significantly modifies the photophysical properties of both porphyrin and protein. We postulate that there are three TSPP binding sites per albumin molecule. To find them in the albumin structure, determine their binding energy, and describe the binding interactions between TSPP and the nearest amino acid residues, we used the Schrödinger software package 2018-1.

## 2. Results

### 2.1. Assessment of Binding Sites from Ground State Absorption Spectra

**Figure 1:**
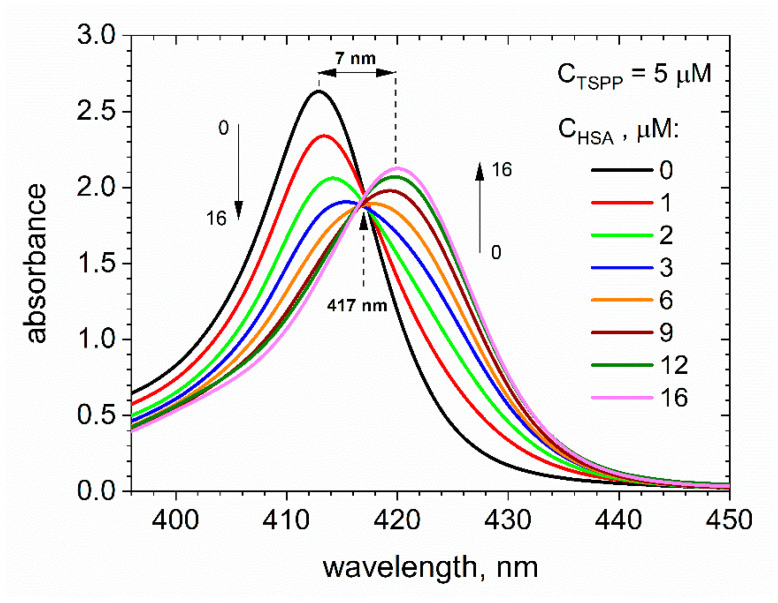
Absorption spectra of TSPP in pH 7 buffer upon addition of different amounts of HSA. Concentration of TSPP is constant and equal to 5.0 µM, HSA concentrations as indicated.

Figure 1 shows the absorption spectra of buffer solutions (pH ca. 7) containing porphyrin with a constant concentration of *C*_TSPP_ = 5 μM and albumin with concentration *C*_HSA_ increasing from 0 to 16 μM. The spectra do not indicate the presence of protonated monomeric or J-aggregated forms of porphyrin, which is consistent with the other reports for neutral porphyrin and porphyrin–HSA solutions [20,24,25]. In the absence of protein, the absorption spectrum of TSPP shows a typical behavior for a monomer with the Soret band, which corresponds to the S_0_→S_2_ transition and has a single peak with a maximum at 412 nm. Upon complexation with albumin, a reduction in the intensity and a redshift of the band are observed. As the HSA concentration increases, a new band with a maximum at 420 nm develops, and this band corresponds to the porphyrin bound to the protein. Around 417 nm, we search for an isosbestic point, but it is not well defined. On the one hand, this indicates the coexistence of the free and HSA-complexed forms of porphyrin in the solution; on the other hand, it suggests that porphyrin can bind to HSA at various sites and the resulting complexes may not have a strict 1:1 stoichiometry [25].

Based on the absorption measurements, we make several attempts to estimate the binding parameters for the TSPP–albumin interaction in a neutral aqueous solution. Our first approach is based on a simple two-site model [27], in which the ligand L (TSPP) can bind to the binding site on a protein P (HSA), according to the equilibrium LP ⇄ L + P with the dissociation constant *K_d_*, as follows:

(1) $$K_{d}=\frac{\left[L][P\right]}{\left[\mathrm{LP}\right]}$$

where [L], [P], and [LP] are the equilibrium concentrations of free TSPP porphyrin, free sites, and the sites of the protein occupied by TSPP, respectively. It is assumed that all the binding sites in a protein are independent and have the same dissociation constant *K_d_*. If each HSA molecule has *n* sites available for accepting TSPP molecules, the following relations occur:

(2) $$nC_{HSA}=\left[P\right]+[LP]$$

(3) $$C_{TSPP}=\left[L\right]+\left[LP\right]$$

where *C*_HSA_ and *C*_TSPP_ are the total concentrations of protein and TSPP in solution, respectively. From Equations (1) to (3), we obtain the following:

(4) $$K_{d}=\frac{\left(C_{TSPP}-\left[LP\right]\right)(nC_{HSA}-\left[LP\right])}{\left[LP\right]}$$

As the intensity of the absorption of TSPP varies significantly on the binding to albumin, we determine the binding parameters, *K_d_* and *n*, as presented below.

The following three reference buffer solutions were prepared: (1) protein-free buffer, (2) 2 μM HSA, and (3) 75 μM has. Another three solutions that differed from the reference solutions due to the addition of porphyrin were prepared as follows: (I) 26 µM TSPP, (II) 2 µM HSA + 26 µM TSPP, and (III) 75 µM HSA + 26 µM TSPP. Then, successive volumes of solutions (I), (II), and (III) were added to the corresponding stock solutions (1), (2), and (3). The addition of solutions with porphyrin to the reference solutions did not change the concentration of HSA. It only increased the concentration of porphyrin. At each stage, for the solutions prepared in this way (three solutions with a gradually increasing TSPP concentration), the absorption spectra were recorded in relation to their respective reference solutions as follows: for (1), it was the buffer itself; for (2), a 2 µM HSA solution; and for (3), a 75 µM HSA. The total porphyrin concentration *C*_TSPP_ in the tested solutions increased from 0.54 to 6.04 μM.

**Figure 2:**
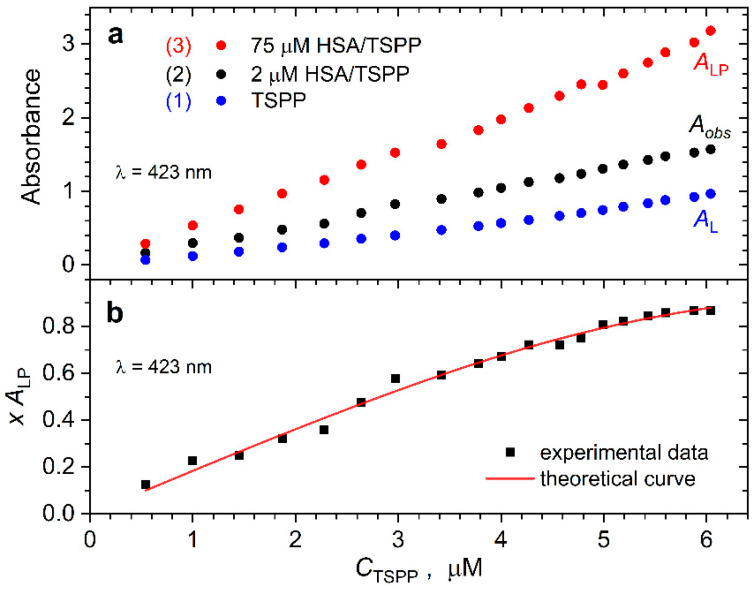
(**a**) Absorbance at λ = 423 nm versus total concentration of porphyrin measured for pH ~7 buffer solutions containing: (1) only TSPP, *A*_L_; (2) 2 μM HSA and TSPP, *A_obs_*; (3) 75 μM HSA and TSPP, *A*_LP_. (**b**) Contribution of the porphyrin in the binding state to the absorbance observed at λ = 423 nm, *xA*_LP_, as a function of *C*_TSPP_ The molar fraction *x* of the binding TSPP molecules is given by Equation (6). The theoretical curve given by Equation (9) is fitted to the experimental data by taking *K_d_*, *n* and *ε_b_* as adjustable parameters.

Figure 2a shows the absorbances *A*_L_, *A_obs_*, and *A*_LP_ collected at λ = 423 nm for solutions (1) to (3), respectively, as functions of *C*_TSPP_. Solution (3) has a significant excess of albumin, so we may assume that almost all the porphyrin added is bound to HSA. Solution (2) has a lower concentration of albumin, so the equilibrium of the binding is determined.

The observed absorbance for this solution, *A_obs_*, can be expressed as the weighted average of the absorbance of the porphyrin in the binding state, *A*_LP_, and in the free state, *A*_L_.

(5) $$A_{obs}=xA_{\mathrm{LP}}+(1-x)A_{L}$$

where *x* is the molar fraction of the binding TSPP molecules. From Equation (5), we obtain the following:

(6) $$x=\frac{A_{obs}-A_{L}}{A_{\mathrm{LP}}-A_{L}}$$

The concentration of porphyrin bound to HSA is equal to [LP] and can be expressed as follows:

(7) $$[\mathrm{LP}]=\frac{xA_{\mathrm{LP}}}{l\varepsilon_{b}}$$

where *l* = 1 cm is the light path of the cuvette and $\varepsilon_{b}$ is the extinction coefficient of bound TSPP. By substituting Equation (7) into Equation (4), we obtain the following equation:

(8) $$K_{d}=\frac{\left(C_{TSPP}l\varepsilon_{b}-xA_{LP}\right)(nC_{HSA}-\frac{xA_{LP}}{l\varepsilon_{b}})}{xA_{LP}}$$

When developing this expression in terms of *xA*_LP_, a quadratic equation is obtained that can be solved and has two possible solutions, as follows:

(9) $$xA_{LP}=0.5l\varepsilon_{b}\left[C_{TSPP}+nC_{HSA}+K_{d}\pm \sqrt{(C_{TSPP}+nC_{HSA}+K_{d}{)}^{2}-4nC_{HSA}C_{TSPP}}\right]$$

From these solutions, only that which is obtained using the minus sign in front of the square root has a physical meaning [28]. Figure 2b shows the experimental data *xA*_LP_ as a function of the total porphyrin concentration in the solution. The solid line is the best-fit curve calculated with Equation (9) using the adjustable parameters *n*, *K_d_*, and *ε_b_*. The values of the parameters obtained are *n* = 2.7 ± 0.2, *K_d_* = 0.3 ± 0.4 μM, and *ε_b_* = 0.20 ± 0.02 μM^−1^cm^−1^, and the coefficient of determination for our fitting procedure is quite high and equals to 0.98.

The results suggest that each HSA molecule should have around three binding sites with a relatively high affinity (binding constant *K_b_* = *K_d_*^−1^ ≈ 3.3 µM^−1^) for TSPP. However, apart from the specific sites which can tightly bind aromatic ligands that are negatively charged on the periphery (like Sudlow site II) [15,29], albumin also has some medium- or low-affinity binding sites. To consider more than one class of binding site, we applied a modified Scatchard formalism [30].

The simple transformation of Equation (4) leads to the following Scatchard equation:

(10) $$\frac{\left[LP\right]}{C_{HSA}[L]}=\frac{n}{K_{d}}-\frac{1}{K_{d}}\frac{\left[LP\right]}{C_{HSA}}$$

which allows for the graphical determination of the binding parameters. This equation shows a linear dependence of [LP]/(*C*_HSA_[L]) on the occupancy of the macromolecule [LP]/*C*_HSA_. Its graph has a slope of −1/*K_d_* and intercepts with the horizontal axis at the abscissa equal to *n*.

**Figure 3:**
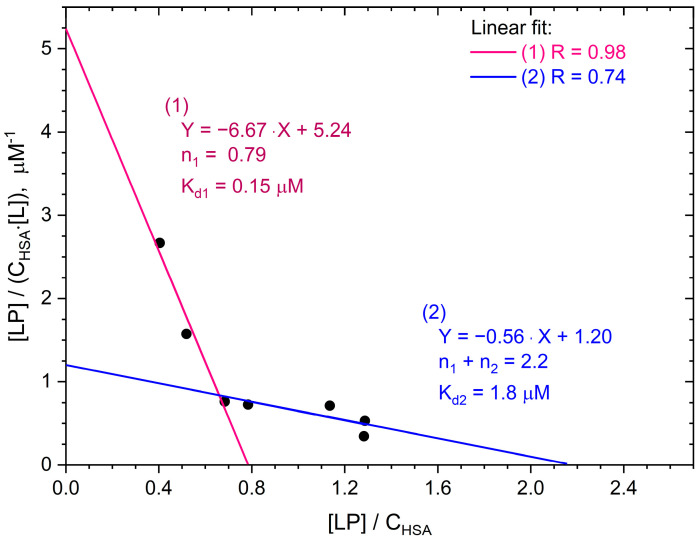
Dependence of [LP]/(*C*_HSA_[L]) on the occupancy of the macromolecule [LP]/*C*_HSA_. [LP] and *C*_HSA_ are concentrations of bound porphyrin and HSA, respectively. Experimental data are calculated using the absorption spectra shown in Figure 1. Assuming that there are two different types of independent TSPP binding sites in the albumin, linear fits for experimental points in the range of low (1) and high (2) values of [LP]/*C*_HSA_ allow for a rough estimation of the binding parameters: *n*_1_, *K_d_*_1_, *n*_2_, and *K_d_*_2_.

Figure 3 shows the dependence of [LP]/(*C*_HSA_[L]) on [LP]/*C*_HSA_ obtained for the HSA–TSPP system based on the experimental data from Figure 1. The concentration of porphyrin bound to HSA was calculated as [LP] = Δ*A*_412nm_/(*l*·*ε*_412nm_), where Δ*A*_412nm_ is the difference in the absorbance measured at *λ* = 412 nm for the TSPP buffer solution (*C*_TSPP_ = 5 μM) in the presence of 1 to 12 μM HSA and TSPP buffer solutions containing no albumin. The extinction coefficient corresponding to the bound porphyrin, *ε*_412nm_ = 257,558 dm^3^·mol^−1^·cm^−1^, was calculated using the Beer–Lambert law, A = ε · c · l, based on absorbance measurements at 412 nm in a buffer solution containing 5 μM TSPP and 16 μM HSA. Free porphyrin concentrations were calculated as [L] = *C*_TSPP_ − [LP]. The relationship shown in Figure 3 (black circles) deviates from linearity. This suggests that the assumption about the existence of one type of identical and independent binding site in a macromolecule, made by Scatchard, is not fulfilled in the case of HSA.

Therefore, we assume that albumin has two types of binding sites with significantly different binding strengths, i.e., *n*_1_ sites with dissociation constant *K_d_*_1_ and *n*_2_ sites with dissociation constant *K_d_*_2_, where *K_d_*_1_ << *K_d_*_2_. If these sites are independent of each other, then the concentration of bound porphyrin is equal to the following:

(11) $$[\mathrm{LP}]=\sum_{i}\left[{\mathrm{LP}}_{i}\right]$$

Furthermore, we can write the following equation similar to Equation (4) for each mode *i*:

(12) $$K_{di}=\frac{\left[L\right](n_{i}C_{HSA}-[LP_{i}])}{\left[LP_{i}\right]}$$

From Equations (11) and (12), we obtain the dependence of [LP] on [L] through the following:

(13) $$\left[LP\right]=\sum_{i}\frac{n_{i}C_{HSA}[L]}{K_{di}+[L]}$$

For two independent classes of binding sites for porphyrin, we have the following equation:

(14) $$\frac{\left[LP\right]}{C_{HSA}[L]}=\frac{n_{1}}{K_{d_{1}}+[L]}+\frac{n_{2}}{K_{d_{2}}+[L]}$$

Equation (14) is analogous to the Scatchard equation. Its interpretation, however, is not as simple as in the case of only one type of binding site. A rough estimation of the porphyrin binding parameters can only be obtained by analyzing the limits of this equation. If the albumin occupancy is low, the concentration of free porphyrin [L] is also low; thus, as [L] goes to zero, we obtain the following:

(15) $$\underset{[L]\to 0}{\lim}\frac{\left[LP\right]}{C_{HSA}[L]}=\frac{n_{1}}{K_{d_{1}}}+\frac{n_{2}}{K_{d_{2}}}$$

A high degree of albumin occupancy can only occur at high concentrations of [L]; thus, as [L] goes to infinity, we obtain the following:

(16) $$\underset{[L]\to \infty }{\lim}\frac{\left[LP\right]}{C_{HSA}[L]}=0$$

However, we must also consider the following:

(17) $$\underset{[L]\to \infty }{\lim}\frac{\left[LP\right]}{C_{HSA}}=\underset{\left[L\right]\to \infty }{lim}\left(\frac{n_{1}\left[L\right]}{K_{d_{1}}+\left[L\right]}+\frac{n_{2}\left[L\right]}{K_{d_{2}}+\left[L\right]}\right)=n_{1}+n_{2}$$

The intersection points of the Scatchard graph with the coordinate axes in the case of two independent types of binding sites can be easily found using the linear fits (1) and (2) shown in Figure 3. Fit (1) refers to experimental points in the range of low albumin occupancy rate, whereas fit (2) refers to points with high [LP]/*C*_HSA_ values. The intersection of the Scatchard graph with the vertical axis is at the point with the ordinate *n*_1_/*K_d_*_1_ + *n*_2_/*K_d_*_2_ (Equation (15)) and with the horizontal axis at the point with the abscissa *n*_1_ + *n*_2_ (Equation (17)). Since *K_d_*_1_ << *K_d_*_2_, the following can be assumed in the first approximation:

(18) $$\frac{n_{1}}{K_{d_{1}}}+\frac{n_{2}}{K_{d_{2}}}\approx \frac{n_{1}}{K_{d_{1}}}$$

(19) $$n_{1}+n_{2}\approx n_{2}$$

It can also be shown that when [L] goes to 0, the slope of the Scatchard curve approaches −1/*K_d_*_1_, and when [L] goes to infinity, the slope of the curve approaches −1/*K_d_*_2_. Thus, the dissociation constants *K_d_*_1_ and *K_d_*_2_ can be estimated from the slopes of line (1) and line (2), in Figure 3, respectively.

Summarizing the above considerations, we can conclude that, assuming the existence of two different types of binding sites in albumin, the estimated values of the TSPP binding parameters are as follows: *n*_1_ = 0.79 ± 0.17, *K_d_*_1_ = 0.15 ± 0.03 µM, *n*_2_ = 2.2 ± 1.5, and *K_d_*_2_ = 1.8 ± 1.1 μM. Thus, the binding constant, *K_b1_,* for the stronger binding sites is equal to 6.6 ± 1.2 µM^−1^, and *K_b_*_2_ for the weaker binding sites is equal to 0.56 ± 0.36 µM^−1^.

### 2.2. Triplet–Triplet (T–T) Absorption Spectra

**Figure 4:**
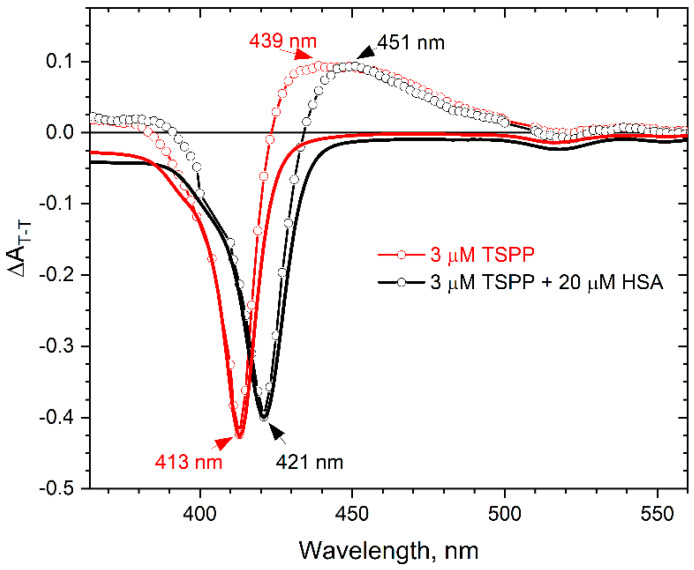
Triplet–triplet (T–T) transient absorption spectra for N_2_-saturated buffer solutions containing 3 µM TSPP in the absence (red circles) and in the presence (black circles) of 20 µM HSA recorded 1.7 µs after a laser light pulse of wavelength 351 nm. The spectrum for the TSPP + HSA solution was multiplied by ~1.5 in order to normalize it to the maximum of the T–T band for the TSPP solution. The solid bold lines correspond to the modified absorption spectra of the considered solutions (original spectrum multiplied by −1 and normalized to the minimum of the corresponding transient absorption spectrum).

We performed laser flash photolysis measurements for the TSPP solutions with and without HSA, and we recorded the triplet–triplet absorption spectra of TSPP, which are shown in Figure 4. As can be seen, the presence of protein clearly changes the T–T absorption spectrum of the porphyrin. The maximum of the T–T absorption for the buffer solution of TSPP is observed at 439 nm, whereas it is observed at 451nm for the TSPP–HSA solution. TSPP transient absorption spectrum has a strong bleaching peak at 413 nm for the buffer solution and at 421 nm for the solution with HSA. The bleached spectra perfectly match the respective absorption spectra of the TSPP and TSPP–HSA solutions, which have been further plotted in Figure 4 (continuous red and black lines) after simple modifications (the absorbance values were multiplied by −1 and then each of the spectra was normalized to the minimum of the corresponding transient absorption spectrum).

These findings, coupled with an analysis of the T–T absorption spectra recorded at longer times (up to 180 μs) after the laser light pulse, indicate that TSPP binds inside the albumin and imply that the excited triplet remains incorporated into the protein and is not expelled to the surrounding solvent during its lifetime. The disappearance of the triplet state is not accompanied by the creation of a new absorption band. Thus, there is no electron transfer between the TSPP and HSA molecules. The T–T decay is simultaneous with the bleaching recovery of the parent absorbance, showing first-order kinetic behavior in both cases, i.e., in the water phase and in the HSA solution. 

**Figure 5:**
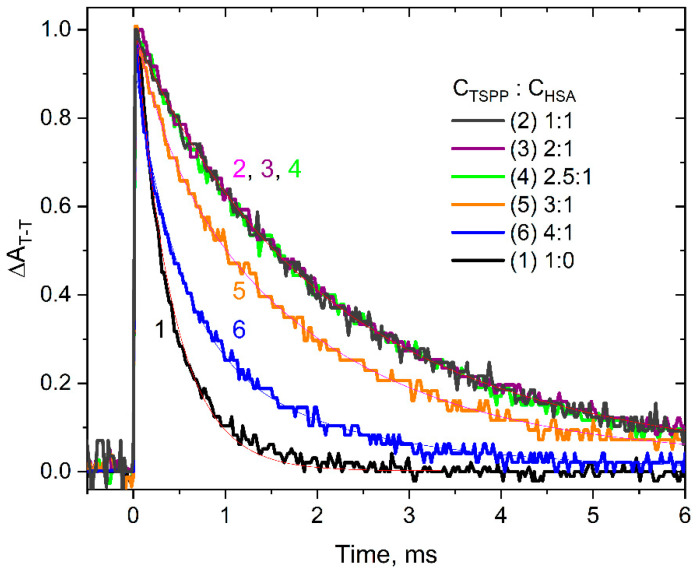
Kinetic curves of triplet–triplet absorbance decay recorded at λ = 460 nm for buffer solutions containing TSPP and HSA in the following molar ratios: 1:0 (10:0 µM; curve 1), 1:1 (30:30 µM; curve 2), 2:1 (40:20 µM; curve 3), 2.5:1 (50:20 µM; curve 4), 3:1 (30:10 µM; curve 5), and 4:1 (40:10 µM; curve 6). Kinetic curves were recorded after a pulse of laser light with a wavelength of λ = 351 nm. The solutions were vacuum deaerated. The kinetic curves were registered for 460 nm. Exponential functions of the best fit are superimposed on the experimental curves. Exponential best-fit functions are superimposed on the experimental curves (parameters of the fits are provided in the Supplementary Materials Table S1).

Figure 5 presents the T–T absorbance decays for vacuum-deaerated solutions containing TSPP and HSA in the following molar ratios: 1:0, 1:1, 2:1, 2.5:1, 3:1, and 4:1. The kinetic curve for the TSPP buffer solution is well described by the monoexponential function, and the decay time of the triplet state equals *t*_1_ = 400 µs. The confined geometry of the porphyrin molecule within the HSA structure restrains the nonradiative decay of the excited triplet state of porphyrin and significantly lengthens its lifetime.

The TSPP molecule encapsulated with HSA gives a triplet lifetime *t*_2_ of close to 2 ms (1.9–2.3 ms). The kinetic curves recorded for the solutions containing TSPP and HSA in molar ratios from 1:1 to 4:1 were fitted using the following biexponential function (Equation (20)), with lifetimes *t*_1_ and *t*_2_ as parameters:

(20) $$∆A_{T-T}\left(t\right)=∆A_{0}+A_{1}\exp\left(-\frac{t}{t_{1}}\right)+A_{2}\exp\left(-\frac{t}{t_{2}}\right)$$

The average decay time of the triplet state of porphyrin *t_av_* [31] for each of these curves was determined according to the following formula:

(21) $$t_{av}=\frac{A_{1}t_{1}^{2}+A_{2}t_{2}^{2}}{A_{1}t_{1}+A_{2}t_{2}}$$

The obtained values of *t_av_* are presented in Table S1 (Supplementary materials). For solutions where the [TSPP]/[HSA] ratio is 2.5 or less, the decay of the TSPP triplet state is described by a single-exponential function (Figure S1 in Supplementary Materials). For the ratio [TSPP]/[HSA] = 3 (or greater), a two-exponential function is necessary to describe the kinetics. In our opinion, this means that there are three porphyrin molecules inside the has, and a small part of the TSPP is in the aqueous phase (a consequence of the binding constant value).

### 2.3. CD Spectra of HSA at Room Temperature

Protein secondary structure can be determined by CD spectroscopy in the far UV spectral region (190–250 nm). Absorption in this region is mostly due to the peptide bond, and the signal arises when it is located in a stable, folded environment. There is a weak but broad n→π* transition around 220 nm (involving the non-bonding electrons of oxygen of the carbonyl group) and a more intense π→π* transition around 190 nm (involving the π-electrons of the carbonyl). 

The α-helix, β-sheet, β-turn, and random coil structures are found in a protein, with each of them giving rise to a characteristic shape and magnitude of CD spectrum in the far UV spectral region.

**Figure 6:**
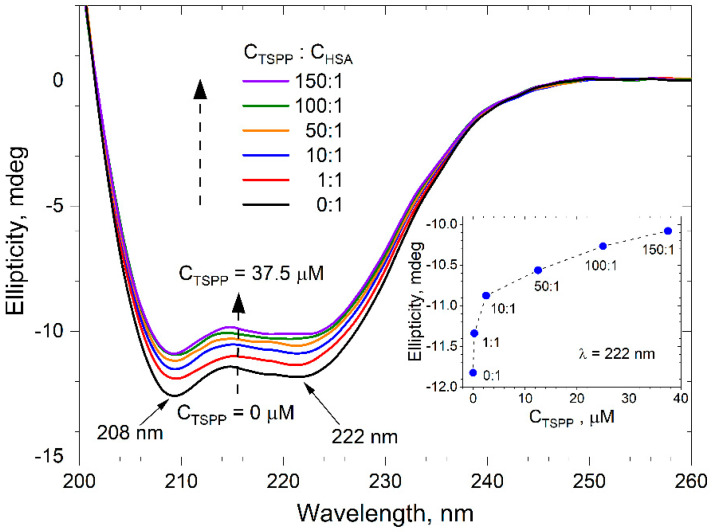
Far UV CD spectra of buffer solution of HSA (0.25 μM) and TSPP; the porphyrin to protein molar ratio, *C*_TSPP_/*C*_HSA_, changes from 0:1 to 150:1, room temperature. Inset: ellipticity measured at *λ* = 222 nm as a function of the porphyrin concentration in the buffer solution of HSA (0.25 μM).

The far UV CD spectra of buffer solutions of HSA (0.25 μM) containing different amounts of TSPP are presented in Figure 6. These negative spectra show two minima, at 208 and 222 nm, which are characteristic of the *α*-helix structure [32]. Upon the addition of increasing amounts of porphyrin, the band intensity of the CD spectra increases without any significant shift of the peaks, indicating that the addition of TSPP changes the secondary structure of HSA.

The secondary structure composition of free albumin, as estimated by the CDNN program, is given in Table 1 and it corresponds quite well with data reported in the literature [33]. This table also shows changes in the composition of the HSA structure caused by an increase in the TSPP concentration.

Increasing the *C*_TSPP_ results in a decreasing tendency of the *α*-helix content and an increasing tendency of the *β*-sheet, *β*-turn, and random coil contents. The *α*-helix remains, however, a predominant conformation of HSA even at molar ratio *C*_TSPP_/*C*_HSA_ = 150:1. A reduction in the *α*-helix structure implies that TSPP binds with the residues of the main polypeptide chain of the albumin and partially destroys the existing hydrogen bond networks. It also indicates that the binding of porphyrin to HSA induces a little unfolding of the polypeptides of albumin, which results in the exposure of some hydrophobic regions that were previously buried.

The inset of Figure 6 presents the dependence of the CD signal, registered at 222 nm wavelength for buffer solutions of HSA (0.25 µM) and TSPP, on the porphyrin concentration. For a molar ratio of porphyrin to albumin lower than 10:1, the ellipticity *θ* decreases very fast with an increase in *C*_TSPP_. A further increase in porphyrin concentration results in a much slower decrease in ellipticity. The arrangement of points on the graph therefore suggests a change in the mechanism of interaction of porphyrin molecules with the secondary structure of the protein.

**Figure 7:**
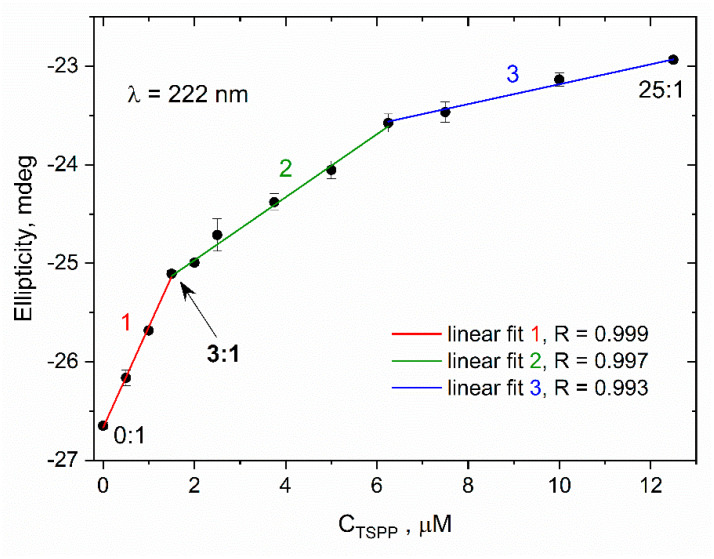
Dependence of ellipticity, registered at *λ* = 222 nm for buffer solutions of HSA (0.5 µM) and TSPP, on the porphyrin concentration, *C*_TSPP_/*C*_HSA_ changes from 0:1 to 25:1, room temperature. Values of ellipticity are obtained as averages of three independent measurements, the error bars are shown in the figure. Data points are fitted by straight lines plotted in the figure, and values of the correlation coefficients *R* are given for each linear fit 1 to 3. The original CD spectra for the data presented in Figure 7 have been added to the Supplementary Material as Figure S2.

In order to estimate the concentration of TSPP at which the nature of protein–porphyrin interaction changes, we made additional measurements of the CD signal for buffer solutions of HSA (0.5 µM) with lower concentrations of porphyrin (up to 12.5 µM). The dependence of ellipticity registered at *λ* = 222 nm on *C*_TSPP_ for the studied solutions is plotted in Figure 7 (the original far UV CD spectra are included in the Supplementary Materials as Figure S1). For these data points, we performed several linear fittings; from them, we chose three regression lines that met the criterion of the best values of the correlation coefficient *R*. These lines, together with the corresponding *R*-values, are shown in Figure 7. 

The first line corresponds to low TSPP concentrations and represents one of the mechanisms of interaction of porphyrin molecules with albumin structure. This mechanism can consist of binding a few consecutive TSPP ligands inside albumin. For intermediate concentrations (the second regression line), other mechanisms or factors become increasingly important, e.g., the electrostatic interaction of ionic porphyrin with the protein surface or the ionic strength of the solution which increases together with *C*_TSPP_. These factors are dominant in the range of high porphyrin concentrations [34,35]. As the C_TSPP_/C_HSA_ ratio increases beyond 12.5 (indicated as the third regression line in Figure 7 and visible in the inset in Figure 6—points above the C_TSPP_:C_HSA_ ratio of 10:1), excess TSPP molecules begin to aggregate independently on the surface of the albumin. In this phase, the interactions between porphyrin molecules become more dominant than the interactions between individual TSPP molecules and HSA. The transition from low to intermediate porphyrin concentration may be associated with a change in the nature of TSPP–HSA interactions. The *C*_TSPP_ value, which determines this transition, was estimated via the intersection of the first and the second regression lines. This value corresponds to the molar ratio *C*_TSPP_/*C*_HSA_ = 3:1.

The CD spectrum of the protein in the near UV spectral region (260–320 nm) can provide some information about its tertiary structure. Absorption in this region results from the presence of aromatic amino acids (π→π* transitions in phenylalanine, tyrosine, and tryptophan residues) and disulfide bonds. The CD signals they produce are sensitive to the overall tertiary structure of the protein [36]. The actual shape and intensity of a protein’s near UV CD spectrum depend on the number of aromatic amino acids of each type contained in this protein, their mobility, the nature of the surrounding microenvironment (hydrogen bonds, polar groups, polarizability) and their distribution in the spatial structure of the protein. In the case of native HSA (pH 7), the spectrum has the minima at 262 and 268 nm and shoulders at 275 and 290 nm [37,38,39].

**Figure 8:**
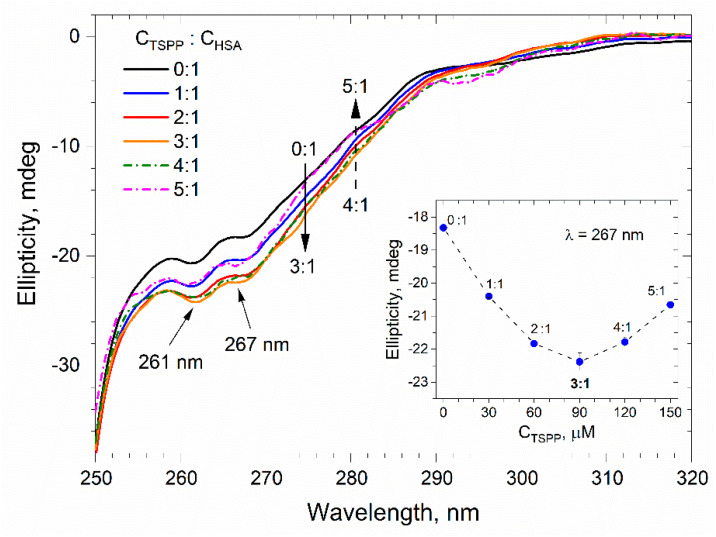
Near UV CD spectra of the buffer solution of HSA (30 µM) and TSPP; the porphyrin to protein molar ratio, *C*_TSPP_/*C*_HSA_, changes from 0:1 to 5:1, room temperature. Inset: ellipticity measured at *λ* = 267 nm as a function of the porphyrin concentration in the buffer solution of HSA.

The CD signal in the near UV range is much weaker than the signal in the far UV range. Therefore, to record the CD spectra in near UV, we prepared solutions with a higher HSA concentration than before. The near UV CD spectra of buffer solutions containing albumin with a concentration of C_HSA_ = 30 µM and porphyrin with a concentration increasing from 0 to 150 µM are presented in Figure 8. The addition of TSPP results in changes in the CD spectrum of albumin. The changes are more pronounced in the spectral region of phenylalanine (255–270 nm) and tyrosine residues (275–282 nm) than in the region of tryptophan residue (285–305 nm) [40]. All these spectra have two minima, at 261 and 267 nm. To determine how strong the influence of the presence of porphyrin has on the tertiary structure of the protein, we made a graph of the dependence of ellipticity recorded at a wavelength of 267 nm on the concentration of TSPP. This graph shows the inset in Figure 8.

As can be seen, with an increase in porphyrin concentration, the CD signal is initially reduced and then, after exceeding the protein to porphiryn molar ratio C_TSPP_/C_HSA_ = 3:1, the CD signal increases. The reduction in CD signal, as a consequence of the protein–porphyrin interaction, indicates the stabilization of the tertiary structure of the protein, while the increase in the CD signal reflects a rearrangement of the tertiary structure of the protein associated with its slight unfolding [41]. It follows, therefore, that a threefold excess of porphyrin in solution optimally increases the folding of the HSA polypeptide chain by forming new stabilizing non-covalent interactions that enhance the HSA structure.

### 2.4. Molecular Docking and Dynamics Simulation

The crystal structures of human albumin used by us in porphyrin molecular docking studies were taken from the PDB database. We present the docking results for two selected crystal structures, i.e., for the native structure of albumin (PDB ID: 1E78) and for albumin crystallized with hemin and fatty acids (PDB ID: 1O9X, Supplementary Materials).

The molecular docking analysis allowed us to identify up to ten conformationally different porphyrin binding sites with different binding energies. Below, we characterize three of these sites for each albumin structure that are considered to bind porphyrin most strongly and are additionally located near the Trp214 residue. Such a choice of binding site locations is due to the experimental results clearly indicating that porphyrin causes a highly effective quenching of the fluorescence of the tryptophan residue of HSA [20]. Since sulfur atoms are effective fluorescence quenchers [42,43], the shortest distance between the S atom of the TSPP side group and the N atom of the tryptophan pyrrole ring is given below as an estimate of the TSPP–tryptophan distance.

#### 2.4.1. Docking and Prime MMGBSA Studies

**Figure 9:**
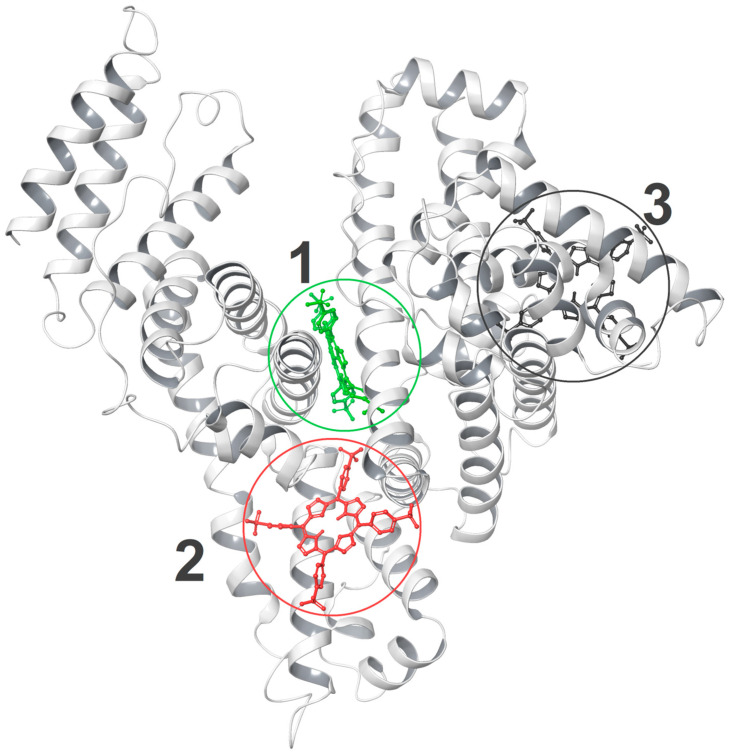
Overall view of the HSA native structure with three TSPP docking sites found, each marked with a circle: green for site 1, red for site 2, and black for site 3.

Figure 9 shows a native HSA structure (PDB ID: 1E78) that does not contain fatty acids or additional ligands, with the locations of the three most effective porphyrin binding sites found in the docking procedure. This HSA structure was chosen due to the fact that the native protein was also used in the experimental studies described above.

To investigate the molecular basis of porphyrin interaction and conformation within the binding sites of the target albumin, we conducted Glide XP docking and Induced Fit Docking (IFD) simulations. The TSPP molecule was found to have the highest binding affinity towards HSA in the first site with a XP GScore of −10.65 kcal/mol, while the XP GScores were −6.86 kcal/mol in the second site and −5.87 kcal/mol in the third. The distances between Trp214 and the porphyrin molecule at the above-mentioned binding sites were 3.53 Å, 5.14 Å, and 19.62 Å, respectively. In order to estimate the free energy of binding (ΔG_bind_) of porphyrin in the protein, we used the Prime MM-GBSA method and obtained a value of −79.28 kcal/mol for the first binding site, −50.66 kcal/mol for the second, and −41.49 kcal/mol for the third site.

**Figure 10:**
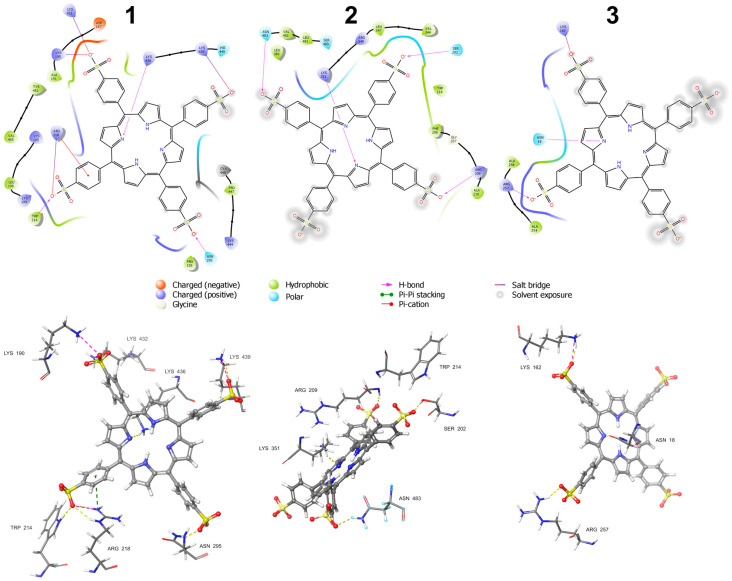
(**Upper Part**): Interactions between TSPP and amino acid residues within three binding sites of HSA (PDB ID: 1E78) illustrated in 2D using the Schrödinger Ligand Interaction Diagram. The three binding sites correspond to those depicted in Figure 9. Residues are depicted as spheres labeled with their respective residue names and numbers, and colored based on their properties (refer to the legend). Interactions between residues and the ligand are visualized as lines, with each interaction type assigned a specific color (refer to the legend). The ligand’s binding pocket is delineated by a colored line surrounding it, indicating the nearest residue’s property. Hydrophobic residues are shown in green, positively charged residues in blue, negatively charged residues in red, and polar residues in cyan. The exposure of the ligand’s atoms to solvent is marked, indicated by a break in the line outlining the pocket. (**Lower Part**): The 3D structure of TSPP interacting with amino acid residues within the binding sites. Different types of interactions are highlighted with distinct colors for the marked bonds: yellow for hydrogen bonds, purple for ionic bonds, and brown for π-cation interactions.

From the docking results, it can be concluded that site 1, where porphyrin is closest to Trp214 (3.53 Å), has the strongest TSPP binding affinity and interacts mainly with the amino acids of subdomains IB, IIA, and IIIA. Structural analysis (see Figure 10) showed that TSPP is stabilized by hydrogen bonds (with Trp214, Asn295, and Lys436), salt bridges (with Lys190, Arg218, Lys432, and Lys439), and π-cation (with Arg218) interactions. At site 2, porphyrin is close to Trp214 (5.14 Å), interacts mainly with the amino acids of subdomains IIA, IIB, and IIIA, and is involved in the hydrogen bond interactions with Ser202, Arg209, Lys351, and Asn483. The third binding site is furthest away from Trp214 and TSPP is stabilized there by interacting with amino acids of subdomains IA, IB, and IIA, by hydrogen bonds (with Asn18, Arg257) and a salt bridge (with Lys162).

It is worth noting that binding sites 1 and 2 are relatively close to the Sudlow site I, and the strong interaction of porphyrin located in these sites with Trp214 may explain the experimentally observed porphyrin-induced quenching of tryptophan fluorescence [24].

The second albumin structure (PDB ID: 1O9X) we considered in the docking simulation was obtained by co-crystallization with hemin (porphyrin derivative) and fatty acids. This structure was chosen for two reasons. First, we wanted to check whether, after the removal of hemin (and fatty acids), the hemin-binding site would also have an affinity for TSPP binding. Secondly, we wanted to compare the TSPP docking results obtained by us using the Schrödinger program with the result obtained by Rozinek et al. [42] for 109X albumin structure from the AutoDock program.

Figure S3 (Supplementary Materials) shows the HSA structure and the locations of the three most effective TSPP binding sites found in the docking procedure. At site 1, TSPP is closest to Trp214 (5.14 Å) and its estimated binding affinity by albumin XP GScore is equal to −8.48 kcal/mol. Structural analysis (Figure S4 in Supplementary Materials) shows that porphyrin is mainly stabilized by π–π interaction with Tyr452, π-cation interaction with Lys190, as well as salt bridges with Lys190, Lys436, and Arg218.

At site 2, porphyrin is also close to Trp214 (5.50 Å) and has a binding affinity with the XP GScore of −5.42 kcal/mol. TSPP interacts mainly with the amino acids of subdomain IIB, namely a π-cation interaction with Lys351, salt bridges with Arg348 and Lys359, and a hydrogen bond with Lys359. It is worth noting that site 2 is located in a similar area of albumin as the binding site found by Rozinek et al. [42]. However, the characteristics of both sites are different. In [42], the TSPP–tryptophan distance was equal to 12 Å, and the electrostatic interactions between the negatively charged TSPP side groups and the positively charged residues (Lys475, Lys205, Arg209, Lys351, and Arg348) were determined as the main stabilizing interactions of TSPP.

Site 3 is located in the IB subdomain and TSPP is stabilized there by interactions with the following residues: Asn18, Asn130, and Lys159 (hydrogen bonds), as well as Lys159 (salt bridge). The XP GScore for this site equals to −6.39 kcal/mol and the TSPP–Trp214 distance is 29.03 Å. It is worth noting that site 3 is significantly shifted from the hemin binding site in the IB subdomain of albumin. Thus, the removal of hemin prior to porphyrin docking does not cause TSPP to bind at a site that was attractive to hemin.

The free energy of binding ΔG_bind_ of porphyrin in the protein calculated using the Prime MM-GBSA method was equal to −58.29 kcal/mol for the first binding site, −58.06 kcal/mol for the second, and −44.89 kcal/mol for the third site.

#### 2.4.2. Molecular Dynamics (MD) Simulations

MD simulations were performed with the application of the Desmond v5.3 software (Schrödinger). The Desmond Interaction Diagrams, which are the result of the analysis of data recorded in the MD simulation trajectory, provided information on the stability of the considered porphyrin–protein complexes under conditions resembling a buffer solution [44]. For a given complex, the first frame of the trajectory corresponded to time *t* = 0 and was treated as the reference frame. The program used the Root Mean Square Deviation RMSD(*t*) function to monitor the average change in the displacement of a selection of atoms for successive frames, i.e., at times t > 0, with respect to the reference frame. All protein frames were first aligned on the reference frame backbone, and then the function RMSD(*t*) was calculated based on the atom selection. In the case of albumin, the selected atoms were the Cα atoms of the amino acid residues. The albumin RMSD functions calculated for all considered systems - did not indicate the occurrence of significant conformational changes and proved that each simulation converged (RMSD values stabilized around a certain mean value). The mean RMSD values for albumin 1E78 in the simulations were in the range of 2.3–2.7 Å for complexes 1–3 and in the range of 3.1–3.3 Å for albumin 1O9X. The selected atoms for the calculation of the porphyrin RMSD(*t*) function were all heavy atoms of TSPP. This function characterizes the stability of porphyrin with respect to albumin and its binding site. The mean RMSD values for porphyrin were in the range of 1.8–5.5 Å. The highest values corresponded to complex 2 in both albumin structures, and this may be related to a temporary loss of stability of the complex (probably as a result of a slight change in conformation, or a slight shift of porphyrin from its initial position). 

**Figure 11:**
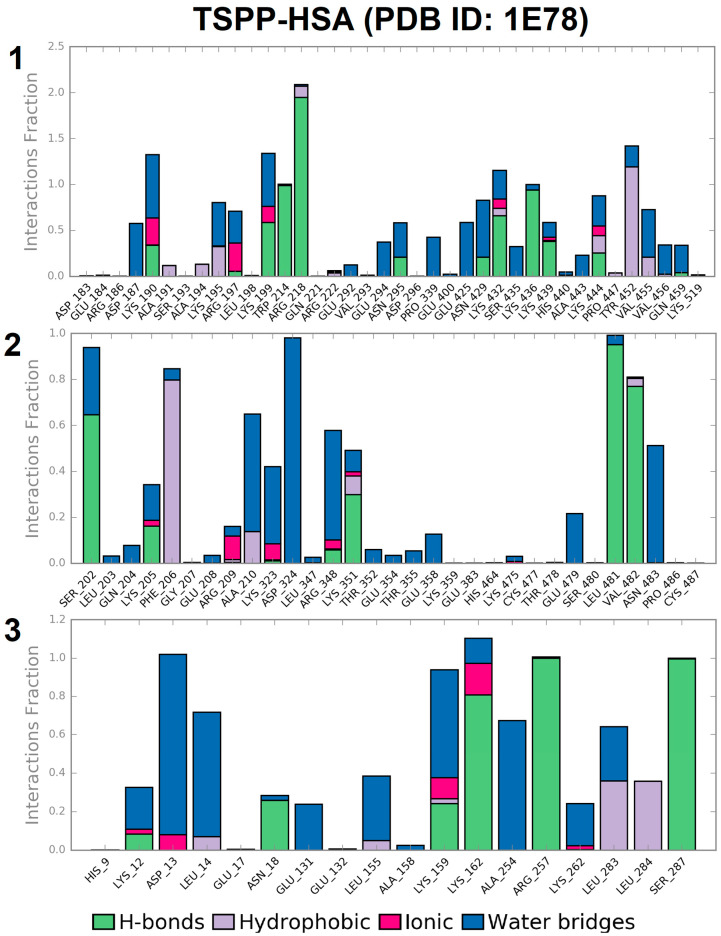
The populations of contacts (interactions) between amino acid residues of albumin (PDB ID: 1E78) and TSPP obtained for the three binding sites during the Desmond MD simulation. The three binding sites correspond to those depicted in Figure 9. Contacts are classified into four types (hydrogen bonds, hydrophobic, ionic, and water bridges) and each interaction type is assigned a color according to the legend.

Albumin interactions with TSPP can be monitored throughout the simulation. The stacked bar charts in Figure 11 show the population of amino acid–porphyrin interactions (or contacts) recorded in each of the considered TSPP–HSA complexes during the 10 ns course of MD simulation. These interactions have been categorized into the following four types: hydrogen bonds, hydrophobic, ionic, and water bridges. The bars shown in Figure 11 reflect the sum of all the interactions in which a given amino acid is involved during the simulation. The charts are normalized over the course of the trajectory; for example, a value of 0.5 indicates that the specific interaction is maintained for 50% of the simulation time. Values above 1.0 are possible because some amino acid residues may form multiple interactions of the same type with the ligand. For instance, two -NH groups of Arg218 form two hydrogen bonds with oxygen of the -SO_3_^−^ TSPP side group. As can be seen in Figure 11, the value of 0.5 for the direct interactions with porphyrin (we ignored the indirect interactions via water bridges) has been exceeded for the following amino acid residues: Lys199, Trp214, Arg218, Lys432, Lys436, Lys444 and Tyr452 for site 1; Ser202, Phe206, Lys351, Leu481 and Val482 for site 2; and Lys162, Arg257, and Ser287 for site 3.

Analogous diagrams prepared for the TSPP–HSA complexes in the 1O9X albumin structure are presented in Figure S5 in the Supplementary Materials. In that case, the porphyrin-stabilizing contacts for more than 50% of the simulation time were interactions with the following amino acid residues: Ala191, Ala194, Arg197, Lys199, Val455, Asn458, and Leu481for site 1; Phe206, Glu208, Arg209, Lys351, and Leu481 for site 2; and Leu135, Lys159, and Lys162 for site 3.

### 2.5. Thermal Unfolding Analysis of HSA

Thermal unfolding and the denaturation of proteins are among the primary causes of protein function loss. Therefore, it is crucial to carefully control and analyze these processes in drug discovery and drug development [45], including sterilization, storage, and other relevant factors. Studying the thermal denaturation of protein in the presence of ligands allows us to determine the effect that they may have on the change (especially increase) in the thermal stability of the protein.

In the tertiary structure of albumin, three domains (I, II, III) are distinguished, and each of them consists of two subdomains (IA, IB, IIA, IIB, IIIA, IIIB). HSA has only one tryptophan residue (Trp214), located in the IIA subdomain [46]. The sequence of thermal unfolding for the three main domains of HSA has been extensively discussed in the literature [41,45,47,48,49,50,51,52]. Despite many studies, this problem remains open because the published results are often contradictory and depend, to a large extent, on the experimental techniques used [47]. Flora et al. [48] proposed a three-step unfolding pathway which started at ~50 °C from reversible separation of domains I and II. Further heating, up to 70 °C, irreversibly unfolded domain II while domain I remained intact. Denaturation of domain I occurred above 70 °C after the completed denaturation of domain II. Their pathways did not include the unfolding of domain III. This domain is considered either as the most unstable and denaturated first, followed by the denaturation of domains I and II [49], or as the most stable domain of HSA [47]. The latter statement does not seem to be correct, however, because other studies [48] have suggested that domain IIIA unfolds reversibly below 55 °C. Farruggia et al. [48] using the Differential Scanning Calorimetry technique found the occurrence of two transitions in the HSA unfolding pathway. The first transition with the midpoint temperature of unfolding (i.e., the temperature at half-completion of the unfolding) Tm = 56.1 °C was interpreted as a paralled unfolding of domains IIA and IIIA (or part of them). The second transition at Tm = 61.6 °C was associated with the unfolding of the part of domain IIA that corresponds to the environment of Trp214.

#### 2.5.1. CD Measurements

**Figure 12:**
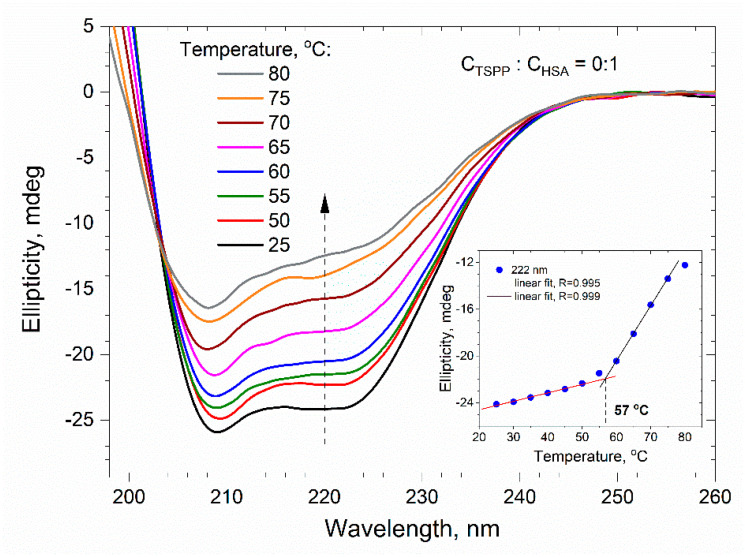
Changes in far UV CD spectrum of the HSA buffer solution (*C*_HSA_ = 0.5 μM) caused by temperature increase from 25 to 80 °C. Inset: ellipticity recorded for wavelength *λ* = 222 nm as a function of temperature. The experimental points are fitted by two straight lines. For each linear fit the value of the correlation coefficient *R* is given.

**Figure 13:**
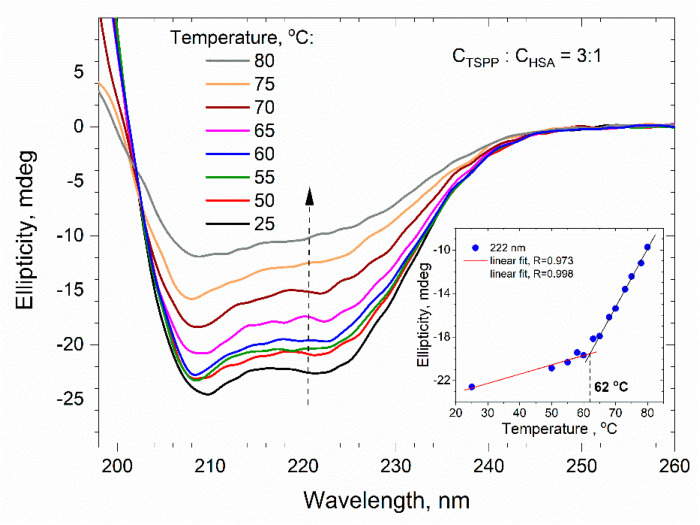
Changes in far UV CD spectrum of the HSA (0.5 µM) and TSPP (1.5 µM) buffer solution caused by temperature increase from 25 to 80 °C. Inset: ellipticity recorded for wavelength *λ* = 222 nm as a function of temperature. The experimental points are fitted by two straight lines. For each linear fit the value of the correlation coefficient *R* is given.

During the thermal unfolding of albumin, the weak intramolecular interactions (e.g., hydrogen bonds) between amino acid residues are gradually destroyed. One of the results of this process is the progressive reduction in the α-helix content in the secondary structure of albumin. To determine the effect of porphyrin on the thermal denaturation of albumin, we measured the far UV CD spectra for a buffer solution of HSA (C_HSA_ = 0.5 μM) without and with TSPP at temperatures varying from 25 to 80 °C. The spectra for the solution without porphyrin are presented in Figure 12, whereas the spectra for the solution containing HSA and TSPP in the molar ratio *C*_TSPP_/*C*_HSA_ = 3:1 are shown in Figure 13. As can be seen, the increase in temperature causes an evident decrease in the intensity of the CD signal resulting from modifications of the secondary structure and the loss of regular folding of the polypeptide chain of albumin. The insets in both figures show graphs of the ellipticity recorded at 222 nm versus temperature. Initially, the ellipticity increases relatively slowly with temperature, but then the increase in ellipticity noticeably accelerates. Both stages of ellipticity growth can be approximated with good accuracy by straight lines, and the intersection of these lines can be used to estimate the temperature at which the mechanism of albumin transition from the native structure to the unfolded structure changes.

The results shown in the insets of Figure 12 and Figure 13 suggest that, for a protein solution without porphyrin, the change in the unfolding mechanism occurs at a temperature of 57 °C. In the presence of porphyrin (at *C*_TSPP_/*C*_HSA_ = 3:1), however, it is shifted to 62 °C.

We can assume that the above-mentioned temperatures determine the beginning of the process of irreversible unfolding of domain II [48] or parallel denaturation of domains IIA and IIIA, which was postulated by the Farruggia group [52]. For the albumin solution, the transition temperature of 57 °C estimated by us agrees well with their temperature Tm = 56.1 °C.

The obtained increase in the denaturation temperature of the protein by about 5 °C, caused by the presence of TSPP, proves that the stability of the secondary structure of the protein is increased by the porphyrin molecules bound to it. The porphyrin–protein complex (in which the porphyrin is located in domain II) requires more energy to weaken the binding interactions between the porphyrin and its surrounding amino acids. During the thermal unfolding of albumin, a certain stabilization of the HSA structure by TSPP also results from the reduction in intramolecular electrostatic repulsive interactions, the mechanism which was well described by Dill [53].

According to the calculations of the CDNN program, when the temperature of the protein solution (0.5 µM) was changed from 25 to 80 °C, the proportion of α-helix in the secondary structure of HSA decreased by 59%. In the presence of TSPP, the proportion of α-helices at molar ratios of *C*_TSPP_/*C*_HSA_ equal of 1:1, 3:1, and 10:1, was reduced to 54.0, 56.4, and 55.8%, respectively.

#### 2.5.2. Differential Scanning Fluorimetry Measurements

To test the thermal stability of HSA, we also applied the differential scanning fluorimetry method (DSF) provided by the Prometheus NT.48 instrument, which monitors intrinsic protein fluorescence changes upon thermal unfolding [54]. In the folded state of HSA, the internal fluorophore Trp214 is buried in the hydrophobic pocket of the protein, where it is shielded from the surrounding aqueous solvent and its fluorescence emission has a maximum of around 330 nm. Upon unfolding, however, the surface of Trp214 becomes exposed and this causes the fluorescence emission peak to shift towards 350 nm. Therefore, the transition of HSA from the folded to the unfolded states can be monitored by registering the changes in Trp214 fluorescence intensity and its emission peak shift [48,51].

Using Prometheus NT.48, we were able to scan the intensity F of tryptophan emission at two different wavelengths, 350 nm and 330 nm, as well as their ratio, F350/F330, while increasing the temperature of the considered protein solution [55].

**Figure 14:**
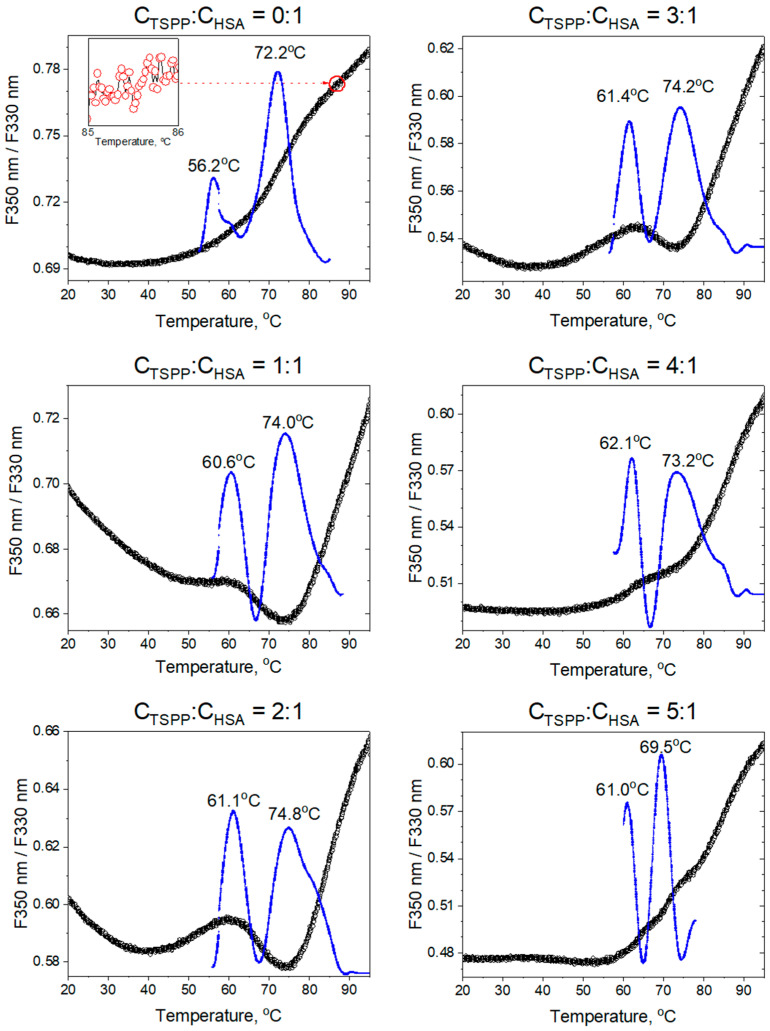
Temperature dependence of fluorescence ratio F350/F330 obtained for buffer solutions of porphyrin and protein in a molar ratio *C*_TSPP_/*C*_HSA_ varying from 0:1 to 5:1 (black circles). The inset in the first picture shows the density of measurement points provided by the Prometheus NT.48 device. The melting (unfolding) temperatures (Tm) for each solution are determined based on the graph of the first derivative of F350 with respect to the temperature. The experimental data are fitted with a polynomial function, and first derivative (blue line) displays peaks at the points of maximal slope, which correspond to Tm.

Measurements were carried out for the buffer solutions containing porphyrin and albumin with the concentration ratios *C*_TSPP_/*C*_HSA_ [μM:μM] equal to 0:1 [0:30], 1:1 [60:60], 2:1 [180:90], 3:1 [360:120], 4:1 [600:150], and 5:1 [900:180]. Figure 14 shows the F350/F330 curve and first derivative of F350, which have been plotted for each solution in the temperature range from 20 °C to 90 °C and 50 °C to 90 °C, respectively. The raw data for F350 and F330 used to determine the F350/F330 ratio are provided in the Supplementary Materials in Figure S6.

The F350 and F330 fluorescence intensities of native albumin decrease with increasing temperature; however, the redshift of the Trp214 fluorescence emission peak accompanying the protein unfolding process causes the F350/F330 ratio to increase with temperature. The fluorescence ratio curve suggests that the unfolding of the native HSA proceeds evenly and starts at a temperature of about 50 °C. However, the first derivative of F350 has two distinct maxima at temperatures Tm1 = 56.2 °C and Tm2 = 72.2 °C, and each of them corresponds to a local acceleration of the protein unfolding process. This may indicate the division of the process into two stages related to the subsequent unfolding of domain II and then domain I [48].

An analysis of the F350/F330 curves for the solutions with molar concentration ratios *C*_TSPP_/*C*_HSA_ from 1:1 to 3:1 indicates that the presence of porphyrin affects the course of the albumin unfolding. All these curves start to rise from a temperature of about 50 °C, have a maximum at around 60 °C, decrease to a clear minimum at 75 °C, and then rise rapidly with further increase in temperature. The albumin domain II begins to unfold around 55 °C. This process causes a change in the environment of tryptophan to a less hydrophobic one and therefore an increase in the F350/F330 emission ratio. Further unfolding of domain II makes it easier for the porphyrin molecules to penetrate inside the albumin. This increases the stability of the domain structure (through the interaction of TSPP molecules with the surrounding amino acids) and changes the tryptophan environment to a more hydrophobic one (by displacing water molecules using TSPP molecules). Lowering the fluorescence ratio above 60 °C is a reflection of these effects. The unfolding of domain I probably starts above 70 °C and is followed by a weakening of the binding forces of TSPP molecules to albumin amino acids. As a result, we observe an acceleration of the destruction of the HSA spatial structure. For solutions where *C*_TSPP_/*C*_HSA_ > 3:1, the F350/F330 curve profiles become smoother and, as in the case of native HSA, do not clearly indicate a multi-stage unfolding.

## 3. Discussion

Potential photosensitizers for use in photodynamic therapy (PDT) and radiotherapy of cancer are of interest to many research groups. The object of our research was 5,10,15,20-tetrakis(4-sulfonatophenyl)-porphyrin (anionic porphyrin, TSPP), the therapeutic efficacy of which in the treatment of some tumors was confirmed by in vitro and/or in vivo tests.

Herein, we extended our previous study [26] by using the absorption measurements to estimate both the porphyrin binding constant, *K_b_*, and the number of binding sites, *n*, in an HSA molecule. The results we obtained, like the results of other researchers, are obviously dependent on the model adopted to describe the protein–ligand system. A similar approach was used in studies on the binding of fluorescent ligands to amyloids. Taylor et al. [56] demonstrated that the pentameric oligothiophene pFTAA binds to amyloids with high affinity. In contrast, Watanabe et al. [57] identified two types of binding sites for chiral proteophene ligands binding to insulin amyloids. Such multi-site analyses highlight the importance of using models that account for different affinities in complex ligand–protein systems, such as TSPP–HSA.

In our study, we considered two models. One of them was a two-state model (free and bound porphyrin) analogous to the model used in the work of Wang et al. [25]. The authors performed picosecond time-resolved emission anisotropy measurements for free TSPP and TSPP–HSA complexes. Based on the results obtained, they concluded that, apart from creating the robust complexes with HSA, some of the porphyrin molecules behaved almost identically to the free molecules in solution. These molecules may form complexes on the surface of the protein or loose-type complexes inside the protein cage.

Although, nowadays, TSPP is recognized as an efficient photosensitizer, there is still a lot of confusion about the number of TSPP molecules that can bind to albumin and the location of TSPP binding sites. There have been several papers in which the binding parameters of TSPP with HSA were estimated. Wang et al. [25] measured the absorption spectra of TSPP in buffer solutions (pH 7) at different concentrations of the albumin. One isosbestic point at 419 nm, which they obtained after plotting these spectra, indicated a 1:1 stoichiometry of the TSPP–HSA complexes. The binding constant of such complexes was estimated as *K_b_* = 1.7 µM^−1^. Similar stoichiometry of the complexes and values of *K_b_* were postulated by An et al. [20] and Andrade et al. [24]. Bartosova et al. [58], however, employed the binding isotherm method and found that, apart from two or three weak binding sites (*K_b_* = 0.2 µM^−1^), albumin also has one strong TSPP binding site (*K_b_* = 3 µM^−1^). Similarly, Ion et al. [59] suggested three binding sites in the albumin molecule, with a binding constant *K_b_* = 1.5 µM^−1^ for one of them and *K_b_* = 0.2 µM^−1^ for the other two. The results of the photochemical and pulse radiolysis measurements reported by Davila et al. [60] suggested that the zinc derivative of sulfonated porphyrin formed complexes with HSA in a ratio greater than 3:1.

Our model assumed the existence of only one type of independent binding site with the same bonding strength and gave a value of *K_b_* = 3.3 µM^−1^, slightly higher than the value obtained by Wang (1.7 µM^−1^). However, the average stoichiometry of the TSPP–HSA complexes predicted by us was 3:1, not 1:1 as in the previous work [25]. More than one binding site per albumin molecule was also postulated by Bartošová et al. [58], Ion et al. [7] and Davila et al. [60]. It is obvious that all these sites cannot have the same affinity for porphyrin. Thus, our next model assumed the existence of two types of independent binding sites with distinct bonding strengths. The values of the binding parameters have been estimated here based on the modified Scatchard formalism. For strongly binding centers, these parameters were equal to *n*_1_ = 0.79 ± 0.17, *K_b_*_1_ = 6.6 ± 1.2 µM^−1^, while for weakly binding sites, *n*_2_ = 2.2 ± 1.5 and *K_b_*_2_ = 0.56 ± 0.36 µM^−1^. The values of *K_b_* seem acceptable because binding constants in the range from 0.01 to 10 µM^−1^ have been reported for porphyrin and albumin complexes [25].

It is worth noting, however, that the assumption of mutual independence of binding sites, which was adopted in our models, may not actually be fulfilled. In biological systems, the binding of the first molecule can change the ability to bind subsequent molecules (i.e., cooperativity). The Scatchard graph obtained for anti-cooperative binding sites is similar to the graph for the model of two types of independent binding sites. Thus, it is practically impossible to distinguish between the two models on the basis of the Scatchard curve.

The laser flash photolysis measurements conducted for the TSPP solutions with and without HSA provided valuable insights. The triplet–triplet absorbance spectra of TSPP demonstrated distinct differences in the presence of HSA compared to the buffer solution. The absorption peaks shifted, indicating binding between TSPP and albumin. Additionally, the bleaching peaks in the transient absorption spectra aligned with the ground absorption spectra, suggesting that the excited triplet state of TSPP remained within the protein and was not expelled into the surrounding solvent. The decay of the triplet state followed first-order kinetics in both the water phase and the HSA solution. Kinetic measurements revealed that the confinement of TSPP within the HSA structure extended the excited-state lifetime of the triplet state. Moreover, by varying the molar ratio of TSPP to HSA, it was observed that the average decay time of the triplet state remained relatively constant up to a ratio of 3:1, indicating binding to three TSPP molecules within the HSA. However, beyond this ratio, the unbound TSPP molecules resulted in a significant shortening of the average triplet lifetime, which is due to the shorter lifetime of the triplet state in the aqueous phase than in HSA. These findings provide valuable information about the interaction and binding of TSPP with HSA, shedding light on the complex behavior of the system.

Andrade et al. [24] performed the CD measurements for the albumin and the TSPP–albumin system with a fixed porphyrin concentration. They recorded the CD signal on far UV and suggested that it largely reflects the secondary structure of the protein and arises from the inherent chirality of the polypeptide chain. Native HSA is a globular protein that contains around 66% of the α-helix conformation at pH 7. The presence of TSPP induced spectral changes, namely a redshift of 4 nm and a decrease in the CD signal, which indicated a certain loss of helicity, presumably caused by electrostatic interactions. The authors [24] did not consider how the CD spectrum would change with the increase in porphyrin concentration. As we were interested in the changes in the albumin secondary structure after the binding of the subsequent porphyrin molecules, we measured the CD spectrum at a constant HSA concentration for various porphyrin concentrations.

Analysis of the far UV CD spectra of albumin in buffer solutions containing varying amounts of TSPP enabled us to determine the porphyrin-induced changes in the secondary structure of HSA. All these spectra had characteristic minima at 208 and 222 nm, indicative of the α-helical structure of protein. The increasing concentration of TSPP resulted in a gradual reduction in these minima without changing their positions. This indicates the conformational change in albumin, that is, a certain decrease in the content of the α-helix structure. We can deduce that the complexation of TSPP disrupts hydrogen bond networks in HSA and causes the partial unfolding of its secondary structure [20]. At the same time, new hydrogen bonds are formed between the nested porphyrin and albumin molecules, contributing to the stabilization of the tertiary structure in the TSPP–HSA complex. This conclusion is confirmed by the results of a molecular docking simulation. It should be emphasized that the porphyrin-dependent reduction in the α-helix structure in albumin is the most rapid when the current concentration of TSPP in solution does not exceed more than three times the concentration of albumin. When we further increased *C*_TSPP_, this process clearly slowed down. It suggests that the molar ratio C_TSPP_/C_HSA_ = 3:1 can be associated with a change in the nature of TSPP–HSA interactions.

In the near UV CD spectra of albumin, porphyrin-induced changes in CD signal were observed primarily in the spectral region of phenylalanine and tyrosine residues and indicated alterations in the tertiary structure of HSA. As the TSPP concentration increased, the CD signal (negative ellipticity) initially decreased; after exceeding the molar ratio C_TSPP_/C_HSA_ = 3:1, it began to increase. This means that low concentrations of porphyrin in solution promote the stabilization of the tertiary structure of albumin, while high concentrations may promote its slight unfolding [16]. Thus, the concentration ratio C_TSPP_/C_HSA_ = 3:1 appears to correspond with the most stable tertiary structure of albumin.

To further explore the structure and properties of porphyrin–HSA complexes, computational modeling offers an insightful approach. So far, the number of papers devoted to computer modeling of aromatic ligand–albumin systems is quite small. Most of them have been published in the last two decades. He et al. [61] performed molecular docking computation to show that guaiacol can bind strongly to HSA, and its primary binding site is Sudlow site I of HSA. A similar calculation for naringin [62] showed that this bioflavonoid is bound in site I in the vicinity of Trp214. This finding provided a good structural basis to explain the efficient fluorescence quenching of HSA emission in the presence of naringin. Molecular docking was also applied to study the binding of isoalantolactone to site I of HSA [63]. The complexation of meso-tetrakis(4-hydroxyphenyl)porphyrin by HSA was considered by An et al. [20], who found that the chromophore can partially enter Sudlow site II of subdomain IIIA via hydrogen bonding and hydrophobic interactions. Rozinek et al. [42] presented the results of molecular docking of TSPP to has that were obtained using the AutoDock computer program. The authors considered only one TSPP binding site in albumin, and the criterion of its selection was not the optimal binding energy of TSPP but its shortest distance from the tryptophan residue.

In our study, the binding interactions between anionic porphyrin and albumin were meticulously explored using Schrödinger software. The TSPP docking procedure was performed for two different albumin crystal structures with the following PDB ID numbers: 1E78 (native structure) and 1O9X (structure co-crystalized with hemin and fatty acids). The main reasons for this choice are given in Section 2.4. In addition, comparing the simulation results for two different albumin input structures seemed interesting. Fatty acids and any additional ligands were removed from both selected structures before the calculations began. For each structure from among the TSPP binding sites indicated by the docking module, we selected three locations for further analysis. The selected sites met the following two criteria: high binding energy and short distance to the rest of Trp214. The latter criterion resulted from the experimentally documented fact of the effective quenching of Trp214 fluorescence by bound porphyrin in HSA.

The numerical characteristics of binding sites in the native albumin structure were as follows: binding affinity in kcal/mol equal to −10.65, −6.86, and −5.87; distance from Trp214 in Å equal to 3.53, 5.14, and 19.62; free energy of binding in kcal/mol (calculated using the Prime MMGBSA module) equal to −79.28, −50.66, and −41.49, respectively, for sites 1, 2, and 3. For the subsequent binding sites 1, 2, and 3 in the 1O9X structure of albumin, the following values were obtained: binding affinity in kcal/mol −8.48, −5.42, and −6.39; distance from Trp214 in Å 5.14, 5.50, and 29.03; and free energy of binding in kcal/mol −58.29, −58.06, and −44.89.

Summing up the above data, we can conclude that the most favorable TSPP binding site we have found is site 1 in the native structure of albumin. A comparison of Figure 9 and Figure S3 suggests that, despite significant differences in the starting albumin structures, the positions of the binding sites 1, 2, and 3 indicated by the docking module in the two structures are similar. However, the immediate vicinity of porphyrin at the corresponding binding sites of the albumin 1E78 and 1O9X is different, and thus interactions with other amino acid residues turn out to be critical for the stability of the resulting complexes (cf. Figure 10 and Figure S3).

In our study, the most favorable binding site for TSPP in the native albumin structure was identified as the first site, which shows similarities to the first binding site proposed by Costa-Tuna et al. [17] for TDFPPS4. Despite minor differences in the ligands, both compounds are anionic sulfonated porphyrins, and the location of the binding site for site 1 appears to be conserved. Our analysis of the molecular docking results showed that TSPP in the first binding site is stabilized by hydrogen bonds with Trp214, which is consistent with the results obtained for the TDFPPS4–HSA complex. Furthermore, in our docking results, interactions of TSPP with Lys190, Arg218, and Asn295 were observed, which correspond to the interactions observed with the same residues in the previous study.

The assessment of dynamic stability of the TSPP–HSA system under conditions resembling a buffer solution was performed using the MD simulation method and the Desmond software. The system information was recorded as the MD trajectory consisting of frames collected every 1.2 ps for the simulation period of 10 ns. Based on these data, calculation of RMSD functions for albumin and porphyrin, as well as an analysis of the interactions shown by porphyrin during the entire simulation, was performed. These results are discussed in Section 2.4.2.

The thermal unfolding analysis of human serum albumin in the presence and absence of TSPP was performed using two different techniques, i.e., CD spectroscopy and differential scanning fluorimetry (DSF). The aim was to understand the effect of TSPP on the thermal stability and the unfolding process of HSA.

CD measurements were conducted to investigate the secondary structure changes in HSA during thermal denaturation. The CD spectra in the far ultraviolet region were recorded for a buffer solution of HSA with and without TSPP, over a temperature range of 25 to 80 °C. The intensity of the CD signal decreased as the temperature increased, indicating the loss of the regular folding of the polypeptide chain in the secondary albumin structure. The unfolding temperature of domain II was estimated to be around 57 °C in the absence of TSPP, while in the presence of TSPP at a molar ratio of C_TSPP_/C_HSA_ = 3:1, domain II unfolded at approximately 62 °C. These results suggest that TSPP increased the stability of the secondary structure of HSA, requiring more energy to weaken the binding interactions between TSPP and the surrounding amino acids. The reduction in α-helix content in the secondary structure of HSA was also observed, with the α-helix share decreasing by 59% in the absence of TSPP, and further decreasing to 54.0%, 56.4%, and 55.8% for the TSPP/HSA molar ratios of 1:1, 3:1, and 10:1, respectively.

In the DSF method, tryptophan fluorescence ratio curves (F350 nm/F330 nm) were used to monitor changes in the peak position and intensity of tryptophan fluorescence which correlated with the fraction of unfolded protein. Analysis of the curves for the TSPP–HSA samples revealed a two-stage unfolding of the HSA structure, namely an increase in the fluorescence ratio from approximately 50 °C to a maximum at around 60 °C, followed by a decrease and a minimum near 75 °C. The unfolding of domain II above 55 °C allowed the TSPP molecules to penetrate the HSA structure, thereby stabilizing domain II. This interaction affected the surroundings of Trp214, resulting in a reduction in fluorescence ratio above 60 °C. Beyond 75 °C, the binding forces between TSPP and the surrounding amino acids were disrupted, leading to the unfolding of HSA and increased fluorescence emission. Thermal unfolding studies of albumin using the Prometheus spectrophotometer demonstrated that native albumin undergoes a two-stage unfolding at temperatures Tm1 = 56.2 °C and Tm2 = 72.2 °C. The addition of porphyrin increased these temperatures by 5 °C and 2 °C, respectively. The unfolding temperatures Tm1 of the HSA domain II resulting from these measurements agree with the values obtained from the CD analysis.

In conclusion, the results obtained from both CD and DSF measurements demonstrated that the presence of TSPP increases the thermal stability of HSA. The findings highlight the potential of TSPP as a modulator of HSA’s structural behavior, providing valuable insights for future drug discovery and development processes.

## 4. Materials and Methods

### 4.1. Materials

The research focused on anionic porphyrin, 5,10,15,20-tetrakis(4-sulfophenyl) porphyrin (TSPP, catalog number 389404), and human serum albumin (HSA, catalog number A1887), which were purchased from Sigma Aldrich, Poznań, Poland. These reagents were used in experiments without further purification. Porphyrin solutions were always prepared immediately before measurement and protected against unnecessary exposure to light. The concentration of porphyrin in the solutions was determined by spectrophotometric method, considering the molar extinction coefficient ε_412 nm_ = 5.30 × 10^5^ M^−1^·cm^−1^ for TSPP [64]. The concentration of HSA was determined spectrophotometrically, assuming the extinction coefficient ε_260 nm_ = 3.57 × 10^4^ M^−1^·cm^−1^ for HSA [65]. Other auxiliary reagents used in the work were of analytical grade purity and were not additionally purified. Doubly distilled water obtained using ion-exchange and Milli-Q Plus system filters (Millipore, Poznań, Poland) was used for the preparation of the solutions, and the measurements (except for those with specified conditions) were conducted at room temperature, i.e., 22 ± 3 °C. The concentration of the phosphate buffer used for preparing buffer solutions with pH ≈ 7 was 10 mM, consisting of a mixture of NaH_2_PO_4_ and Na_2_HPO_4_, along with 0.17 M NaCl. For measurements conducted under anaerobic conditions, special cuvettes and a vacuum pump were used.

### 4.2. Absorption Spectrophotometry

UV–VIS absorption spectrophotometric measurements were performed using Varian spectrophotometers (Cary 5E model). The linearity of the instruments should be maintained up to an absorbance of 3, so the concentrations of the tested samples were adjusted to ensure that this absorbance value was not exceeded. Absorption spectra were recorded with a resolution of 0.5 nm.

### 4.3. Flash Photolysis

For measurements allowing for the registration of decay or formation of triplet states, a Lambda Physik COMPex 201 excimer laser was used. The emission line at 351 nm (XeF) was used, with a pulse duration of 22 ns and a maximum energy of 300 mJ for the XeF mixture. Since the pulse energy was sufficiently high to cause ionization or interaction between excited states of the compound (mainly triplet–triplet interactions), a filter reducing the laser radiation energy by approximately 11 times was used in the measurements. The size of the laser beam was 3 × 1 cm, and the measuring cuvette had an optical path of 1 cm × 1 cm, which gave the energy absorbed by the studied solution the order of 9 mJ.

### 4.4. Circular Dichroism, CD

CD measurements were performed in the far and near UV range using a Jasco J-815 spectrometer equipped with a thermoelectrically controlled cuvette holder. For measurements in the far UV range, the initial HSA solution (100 μM in phosphate buffer) was diluted to a concentration of 0.5 μM, and then a concentrated TSPP solution was added to achieve final porphyrin concentrations of 0.25–37.5 μM (the concentration of HSA in these prepared solutions remained practically unchanged). CD spectra were measured in the range of 195 to 260 nm in quartz cuvettes with an optical path length of 0.5 cm (Hellma). For measurements in the near UV range, an HSA solution with a concentration of 30 μM was used, and then TSPP porphyrin was introduced into it at concentrations of 30–150 μM. CD spectra were measured in the range of 250 to 320 nm in quartz cuvettes with an optical path length of 1 cm (Hellma). The following measurement parameters were set: light beam slit width was 1 nm; instrument response time was 1 s; scan speed was 50 nm/min; registration of a single CD spectrum was conducted every 1 nm; and the number of CD spectrum measurements for each sample ranged from two to three. The CD spectra obtained for the HSA and HSA–TSPP solutions were corrected by subtracting the CD spectra obtained for the phosphate buffer and TSPP solution in this buffer, respectively. The CD signal for the buffer and the buffer with TSPP (0.25–150 μM) did not exceed ±1 mdeg. Simultaneously with the CD signal, the HT (high tension) signal was measured. In all the presented CD results, the HT signal was below 600 V. The CD spectra deconvolution software CDNN 2.1 was used to determine the percentage content of secondary structures in albumin.

### 4.5. Differential Scanning Fluorimetry 

The concentrations of HSA and TSPP used in the measurements were provided in the section describing the research results. The experiment used a buffer composed of 50 mM HEPES buffer at pH 7.4, 3 mM MgCl2, 3 mM MnCl2, 1 mM DDT, and 0.05% Tween-20. The measurement solutions were placed in UV capillaries (NanoTemper Technologies, Kraków, Poland), and the measurements were performed using the NanoTemper Prometheus NT.48 device and repeated three times for each sample. The temperature gradient was set at 0.5 °C/minute in the temperature range of 20 to 90 °C. Changes in the fluorescence of the protein’s internal fluorophore (tryptophan) occurring during the thermal unfolding process were measured at emission wavelengths of 330 nm and 350 nm (positions of the maximum tryptophan fluorescence intensity for native and denatured protein, respectively) with excitation at 280 nm. The protein unfolding temperature (Tm) was determined by analyzing the fluorescence intensity curves at 330 nm and 350 nm as a function of temperature or, alternatively, by analyzing the fluorescence intensity ratio curves (F350/F330) as a function of temperature. The Tm values were determined based on the maxima of the first derivative of the fluorescence intensity (F350) with respect to temperature.

To study the thermally induced unfolding of HSA, we used the DSF technique and did not use a classic spectrofluorimeter for two reasons. The first reason is trivial and related to the time of measurements, which are much more time-consuming when using a spectrofluorimeter combined with thermostatting of a classic water bath. The second reason for the use of the DSF device is related to its design, namely the use of thin capillaries instead of 1 × 1 cm cuvettes. This allows for a partial compensation of the inner filter effect associated with the high light absorption by porphyrin.

### 4.6. Computational Modeling

#### 4.6.1. Ligand Preparation

The 3D structure of the TSPP4 anion was modeled in GaussView 5.0 [66] and optimized in Gaussian 09 [67]. The particle geometry was optimized by the DFT method using the B3LYP correlation–exchange and 6–31G ++ (d, p) functional bases. The DFT calculations were performed for the continuous PCM solvent model. The optimized ionic porphyrin structures, together with the generated charge distribution (determining the electrostatic potential of the molecule), were saved in .mol2 format using Babel software v2.4.1 [68] and transferred to the Schrödinger program as a ready-to-dock structure in HSA.

#### 4.6.2. Protein Preparation

The 3D X-ray crystal structures of human serum albumin (HSA) complexed with or without ligands were obtained from the Protein Data Bank (PDB) with access codes 1O9X and 1E78, respectively. The collected albumin structures were prepared in a multi-stage process to increase the accuracy of the structure offered by the Schrödinger’s Protein Preparation Wizard module (Maestro software, v11.8) [69,70]. The process removed all water molecules and ligands and optimized the network of hydrogen bonds. Additionally, the energy of the entire system was minimized using the OPLS3 force field. The optimized albumin structures were subjected to SiteMap software, v4.6.011, which searched for potential ligand binding sites.

#### 4.6.3. Docking and MD Simulation

Porphyrin was docked using the Glide module (Ligand Docking function, Schrödinger). The process uses semi-flexible docking, in which the conformation of the receptor is unchanged, while the ligand molecule undergoes translations, rotations, and conformational changes [71]. XP precision docking (extra precision docking and scoring) was employed, allowing for ten ligand configurations to be obtained at the binding site and minimizing ligand energy after docking.

In the next step, the Induced-Fit Docking (IFD) calculations were performed for a standard protocol using the OPLS3 force field and the GBSA solvent model. Ligands docked at the binding sites in the HSA structure were obtained. IFD iteratively combines the technique of ligand docking onto a rigid receptor, with the technique of modeling conformational changes of the receptor. It uses the Glide docking program for ligand flexibility and the Refinement module (Prime software, v3.000) for receptor flexibility. The Prime MMGBSA approach was used to determine the free energy of binding for the ligand–receptor complex.

The obtained ligand–receptor complexes were simulated with the MD method using the Desmond module [72]. The System Builder utility in Desmond generated a simulation box surrounding the entire protein, along with a 10 Å wide margin of space around the protein, which was filled with explicit water molecules described by the SPC model. The system was neutralized, and a 0.15 M concentration of NaCl was added. Calculations were carried out in a standard way using the OPLS3 force field. Before the actual MD simulation began, the system was subjected to a molecular relaxation procedure consisting of several energy minimizations and then short MD runs (12 to 24 ps), with a gradual increase in the temperature of the system up to the assumed simulation temperature. The production time for MD was set to 10 ns, with a time step of 2 fs, for a temperature of 300 K and a pressure of 1.013525 bar. The frame recording interval was set to 1.2 ps. The trajectories obtained were then analyzed using the Simulation Interactions Diagram utility in Desmond.

The MD method enables a more effective elimination of the steric collisions and stresses that appear at the docking stage thus allowing for more considerable conformational changes to be modeled than in the case of the previously described methods based on the sampling of the conformational space of the protein and ligand.

## 5. Conclusions

In conclusion, the research conducted on the interaction between 5,10,15,20-tetrakis(4-sulfonatophenyl)-porphyrin (TSPP) and human serum albumin (HSA) revealed important insights into the binding parameters, structural changes, and thermal stability of the TSPP–HSA complex. The study confirmed the existence of multiple binding sites in the albumin structure with varying affinities for TSPP. Techniques such as UV–VIS absorption, laser flash photolysis, circular dichroism, and differential scanning fluorimetry provided evidence of the porphyrin–albumin interaction that highlighted the resulting changes in absorption spectra, fluorescence intensity, and protein secondary and tertiary structures. The computational analysis using molecular docking and molecular dynamics simulations supported the experimental findings by identifying the specific binding sites and amino acid interactions involved in the stabilization process of the TSPP–HSA complex. Overall, these findings contribute to the understanding of the behavior of TSPP in the presence of HSA and thus have implications for the development of photodynamic therapy and cancer treatment methods. 

## Figures and Tables

**Table 1:** The content of different secondary structures of free HSA and TSPP–HSA systems (CD spectra) at pH 7.2.

Molar Ratio [TSPP]:[HSA]	*α*-Helix %	*β*-Antiparallel %	*β*-Sheet %	*β*-Turn %	Random Coil %
0:1	50.1 ± 0.7	4.3 ± 0.2	6.3 ± 0.1	14.8 ± 0.1	25.1 ± 0.3
1:1	47.4 ± 0.7	5.2 ± 0.2	6.4 ± 0.1	15.1 ± 0.2	26.0 ± 0.2
10:1	45.4 ± 0.9	5.9 ± 0.4	6.5 ± 0.1	15.4 ± 0.1	26.5 ± 0.2
50:1	43.9 ± 0.7	6.5 ± 0.4	6.6 ± 0.1	15.5 ± 0.1	26.9 ± 0.1
100:1	42.4 ± 0.2	7.0 ± 0.3	6.6 ± 0.1	15.8 ± 0.1	27.7 ± 0.5
150:1	42.4 ± 0.2	7.2 ± 0.7	6.6 ± 0.0	15.9 ± 0.4	27.4 ± 0.8

## Data Availability

All data generated or analyzed during this study are included in this published article. T.S. should be contacted if someone wishes to make a request for the data.

## Supplementary Materials

The following supporting information can be downloaded at: https://www.mdpi.com/article/10.3390/ijms252212473/s1.
