# Supplementary material for: Multi-Spectroscopic and Molecular Modeling Studies of Interactions Between Anionic Porphyrin and Human Serum Albumin
Source: Int J Mol Sci. 2024 Nov 20;25(22):12473. doi: 10.3390/ijms252212473 (PMC11595176; doi:10.3390/ijms252212473)
Supplement: Supplementary file 1 [file ijms-25-12473-s001.zip › ijms-3255447-supplementary.pdf]

Supplemental Figures and Tables

Table S1. Parameters of exponential fits for kinetic curves of triplet-triplet absorbance decay for buffer solutions containing TSPP and HSA at various molar ratios. The kinetic curves were recorded at λ = 460 nm following a laser pulse at λ = 351 nm. The solutions were vacuum-deaerated. In the upper section of the table, results are shown for **Figure 5** without time anchoring (data for a two-exponential spontaneous fit without time anchoring). The middle section contains results with time anchoring, including fits for a 400-microsecond triplet decay time in buffer and one for 1900 microseconds. In the lower section, additional results are presented for this system that were not shown in **Figure 5**.

	**TSPP:HSA ratio**	**TSPP:HSA concentration**	R^2	t_{av}, ms	A_1	t_1, ms	A_2	t_2, ms
curve 1	1:0	10 μM TSPP : 0 μM HSA	0.9967	-	1	0.40	-	-
curve 2	1:1	30 μM TSPP : 30 μM HSA	0.9984	2.27	0.0768	0.27	0.9359	2.29
curve 3	2:1	40 μM TSPP : 20 μM HSA	0.9964	2.36	0.1034	0.78	0.8786	2.42
curve 4	2.5:1	50 μM TSPP : 20 μM HSA	0.9978	2.37	0.0860	0.29	0.9321	2.39
curve 5	3:1	30 μM TSPP : 10 μM HSA	0.9958	1.90	0.2545	0.40	0.7512	2.00
curve 6	4:1	40 μM TSPP : 10 μM HSA	0.9967	1.41	0.6650	0.52	0.2620	2.00
curve 2	1:1	30 μM TSPP : 30 μM HSA	0.9983	2.31	0.0705	**0.40**	0.9294	2.33
curve 3	2:1	40 μM TSPP : 20 μM HSA	0.9953	2.29	0.0435	**0.40**	0.8936	2.31
curve 4	2.5:1	50 μM TSPP : 20 μM HSA	0.9978	2.39	0.0806	**0.40**	0.9060	2.42
curve 5	3:1	30 μM TSPP : 10 μM HSA	0.9966	1.84	0.1900	**0.40**	0.8096	1.91
curve 6	4:1	40 μM TSPP : 10 μM HSA	0.9931	1.14	0.5237	**0.40**	0.4763	1.38
curve 6	4:1	40 μM TSPP : 10 μM HSA	0.9920	1.37	0.7292	0.50	0.3157	**1.90**

not shown	1:1	15 μM TSPP :<br>15 μM HSA	-	2.39	0.1779	0.40	0.8220	2.46
not shown	1.7:1	50 μM TSPP :<br>30 μM HSA	-	2.33	0.2086	0.82	0.7914	2.46
not shown	3:1	45 μM TSPP :<br>15 μM HSA	-	1.87	0.2063	0.13	0.7937	1.90

Abbreviations: R – correlation coefficient of the fit; t_{av} – average lifetime; A_1, A_2 – amplitudes of the fit components; t_1, t_2 – lifetimes for each exponential component. The bold number indicates a fixed value, the decay time of the TSPP triplet state in the buffer solution is 400 μs or 1900 μs time of ^{3*}TSPP within HSA.

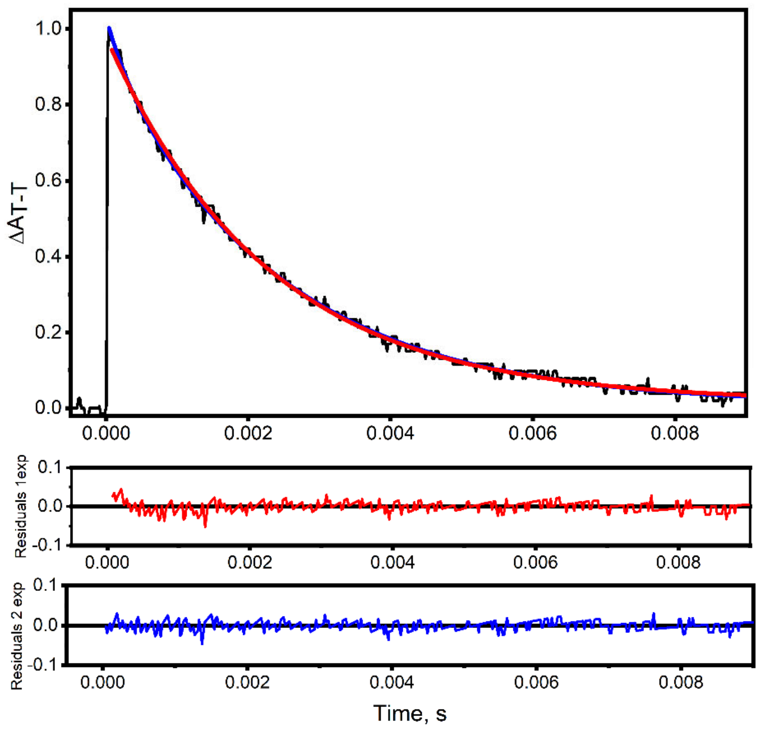

Figure S1. Decay of TSPP triplet state in the presence of HSA (black line) and biexponential best fit to experimental curve (smooth red line) and monoexponential fit (smooth blue line). Panels below present a random distribution of residuals. The concentration of porphyrin is equal to 50 μM and HSA is equal to 20 μM. Triplet-triplet absorbance decay recorded at λ = 460 nm.

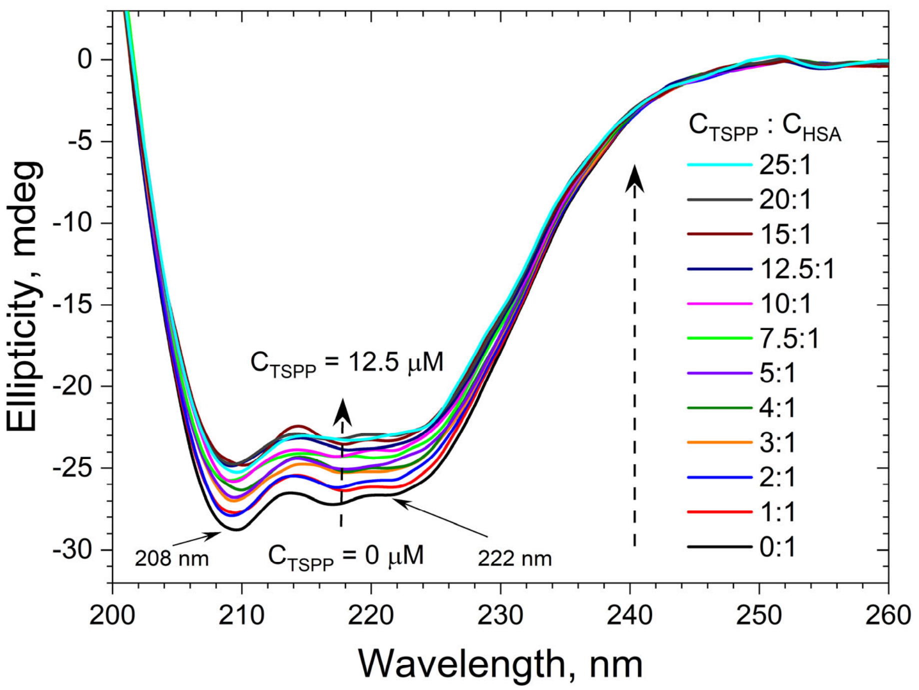

Figure S2. Far UV CD spectra of buffer solution of HSA (0.5 μM) and TSPP; the porphyrin to protein molar ratio, CTSPP:CHSA, changes from 0:1 to 25:1, T = 298 K.

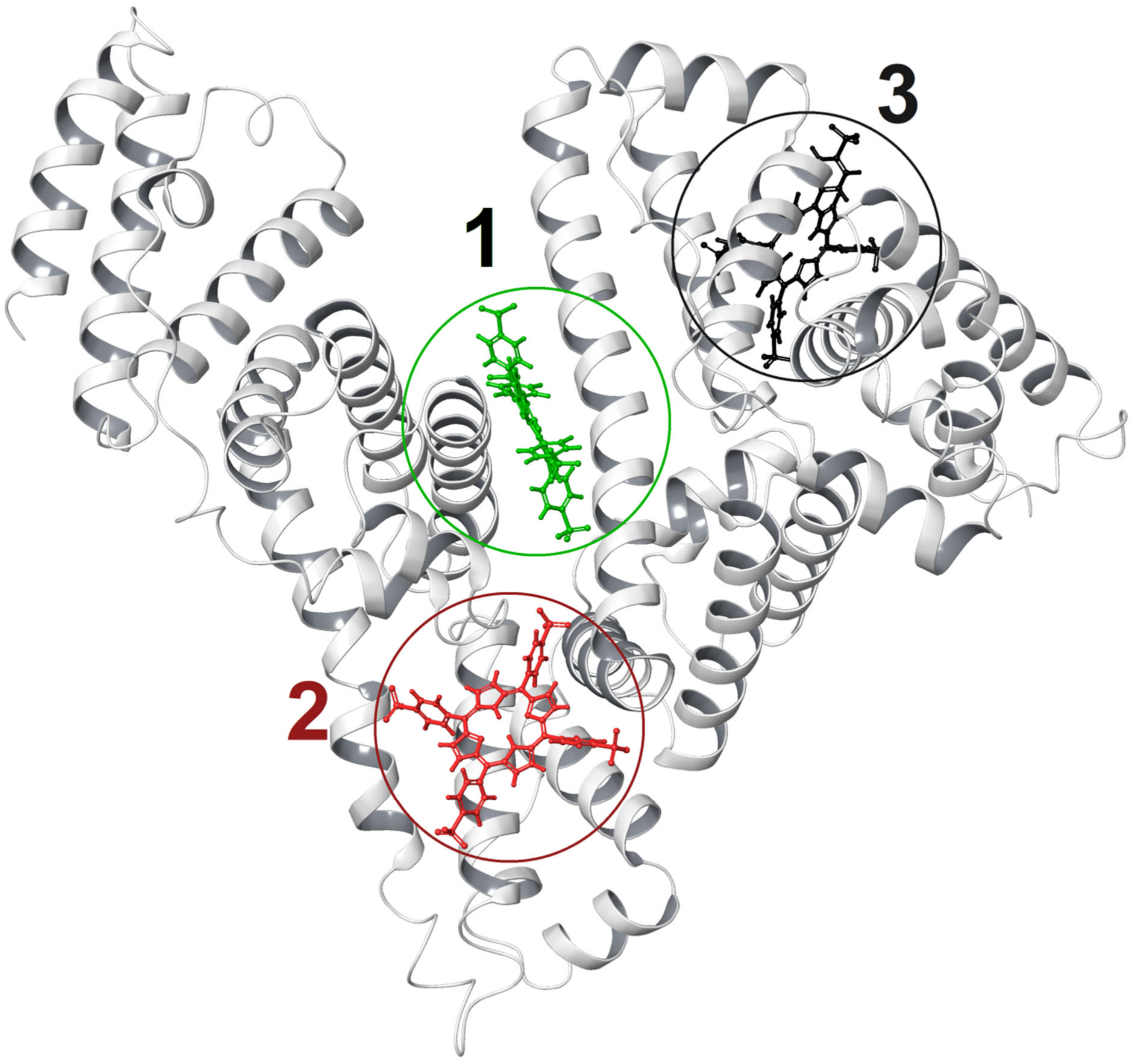

Figure S3. Overall view of HSA structure (PDB ID: 1O9X) with three TSPP docking sites found, each marked with a circle: green for site 1, red for site 2, and black for site 3.

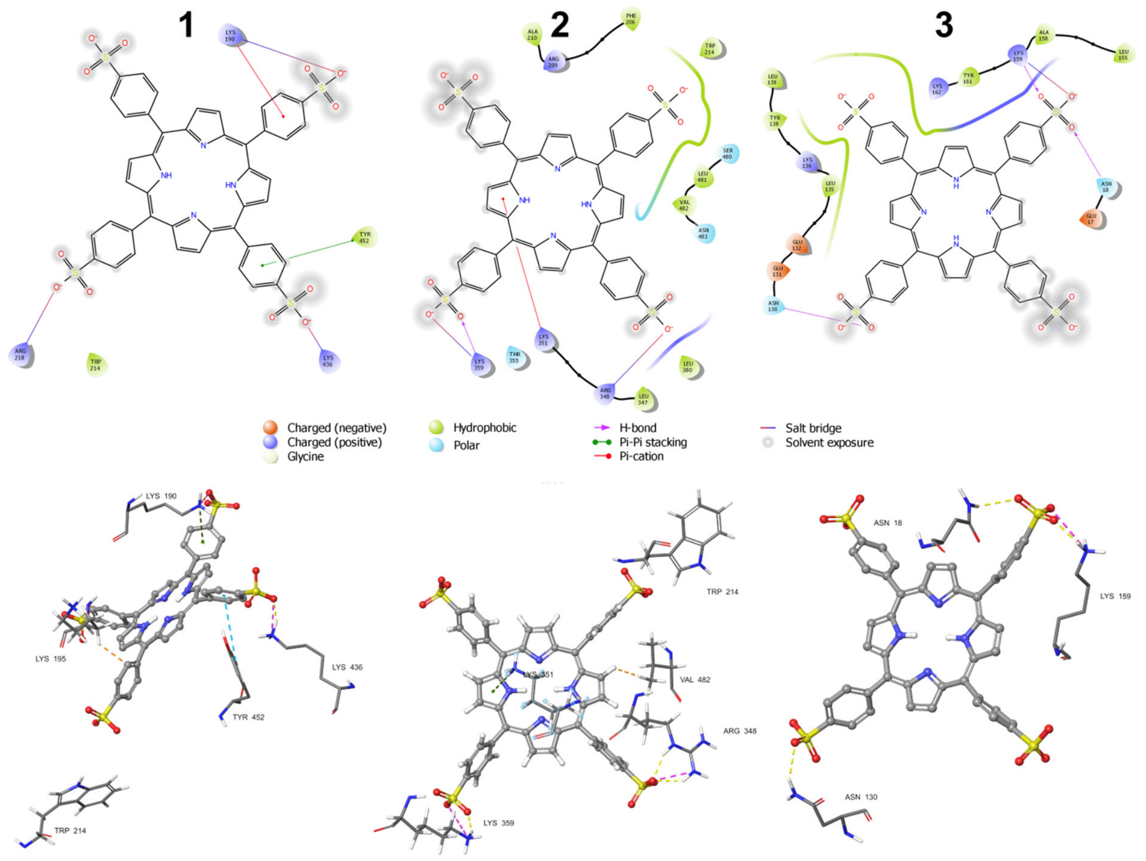

Figure S4. Upper Part: Interactions between TSPP and amino acid residues within three binding sites of HSA (PDB ID: 1O9X) are illustrated in 2D using the Schrödinger Ligand Interaction Diagram. Residues are depicted as colored spheres, labeled with their respective residue names and numbers, and colored based on their properties. Interactions between residues and the ligand are visualized as lines, with each interaction type assigned a specific color (refer to the legend). The ligand's binding pocket is delineated by a colored line surrounding it, indicating the nearest residue's property. Hydrophobic residues are shown in green, positively charged residues in blue, negatively charged residues in red, and polar residues in cyan. The exposure of the ligand's atoms to solvent is marked, indicated by a break in the line outlining the pocket. **Lower Part:** The 3D structure of TSPP interacting with amino acid residues within the binding sites. Different types of interactions are highlighted with distinct colors for the marked bonds: yellow for hydrogen bonds, purple for ionic bonds, and brown for π-cation interactions.

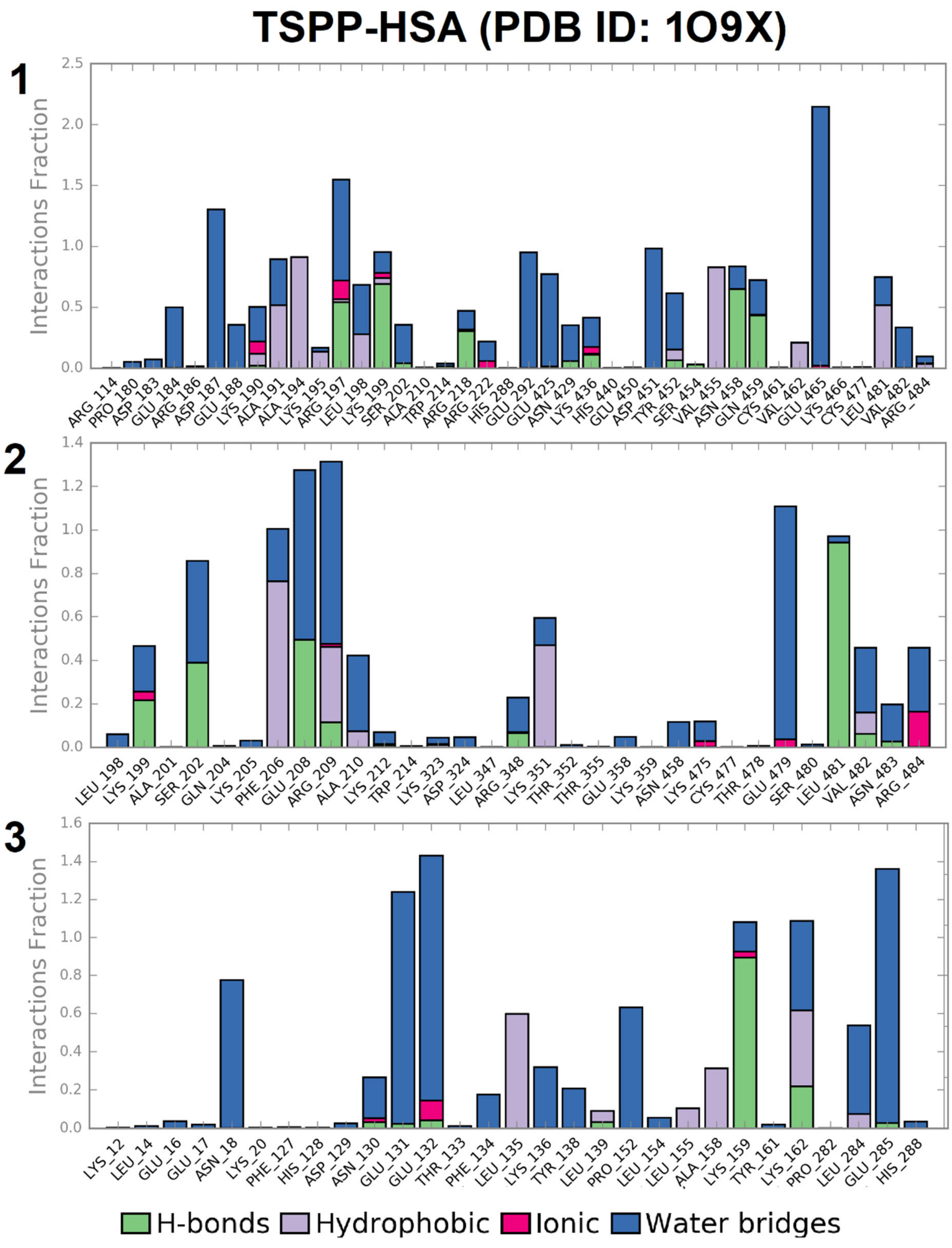

Figure S5. The populations of contacts (interactions) between amino acid residues of albumin (PDB ID: 1O9X) and TSPP obtained for the three binding sites during the Desmond MD simulation. Contacts are classified into four types (hydrogen bonds, hydrophobic, ionic, and water bridges) and each interaction type is assigned a color according to the legend.

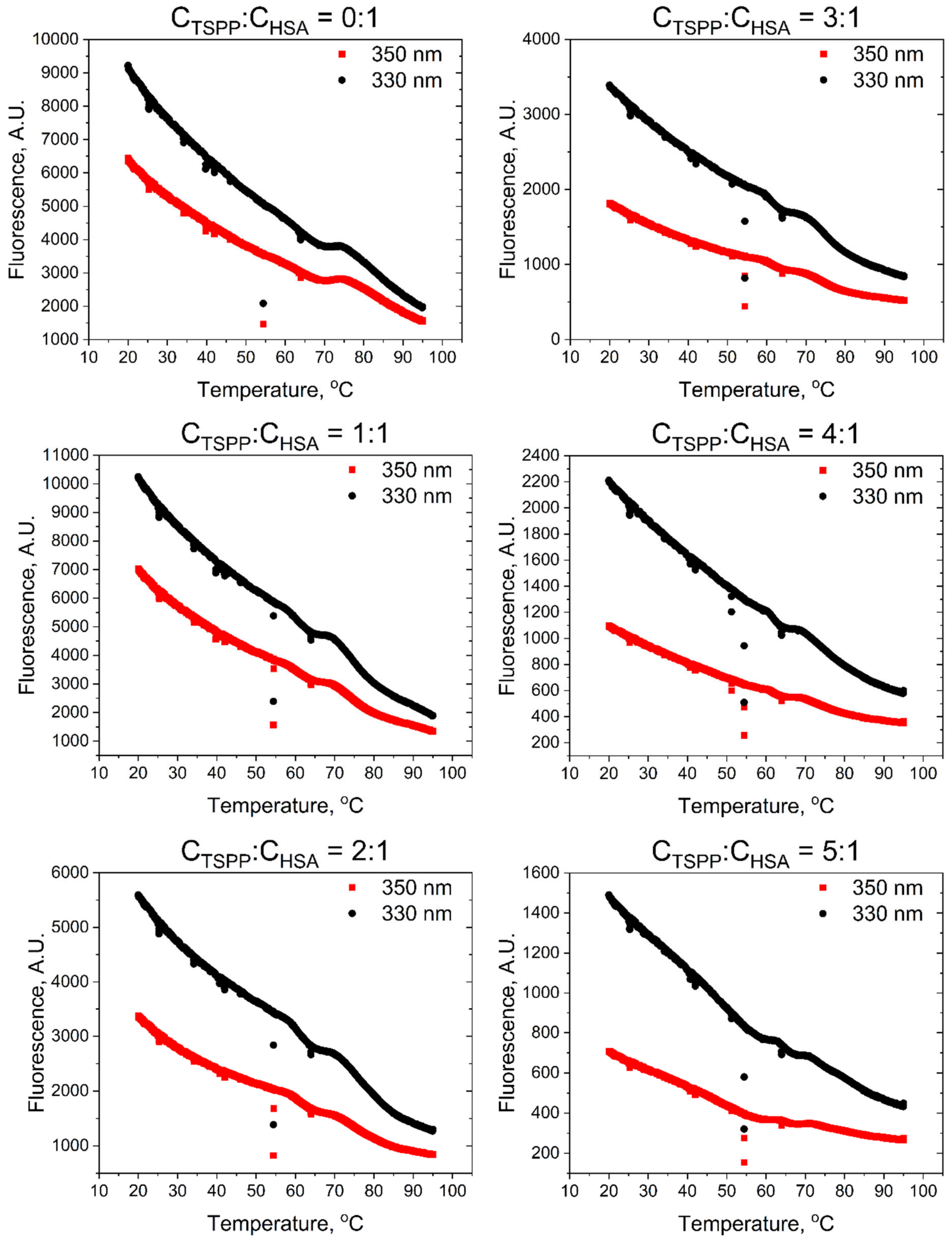

Figure S6. Raw fluorescence data (F350 and F330) used to calculate the fluorescence ratio F350/F330 presented in Figure 14. Each plot shows the temperature dependence of fluorescence intensities F350 and F330 for buffer solutions of porphyrin and human serum albumin (HSA) in molar ratios C_{TSPP}:C_{HSA} varying from 0:1 to 5:1.
